# Integrative multi-omics identifies coordinated alterations in the gut microbiome and in plasma and aqueous humor metabolomes in high myopic cataract

**DOI:** 10.1038/s41598-025-23265-8

**Published:** 2025-11-12

**Authors:** Xiaoxun Gu, Xiaoting Ruan, Wen Yang, Jing He, Li Tang, Dongling Niu, Bo Ma

**Affiliations:** 1 Department of Ophthalmology, Department of Laboratory Medicine, Xi’an People’s Hospital (Xi’an Fourth Hospital), Shaanxi Eye Hospital, Affiliated People’s Hospital of Northwest University, Jiefang Road 21, Xi’an, 710004 Shaanxi China; 2 Department of Ophthalmology, Yantai Yuhuangding Hospital, Qingdao University, Yantai, Shandong China

**Keywords:** High myopia, Gut microbiome, Plasma metabolome, Aqueous humor metabolome, Axial lengths, Biochemistry, Biomarkers, Diseases, Microbiology

## Abstract

Growing evidence suggests that chronic inflammation contributes to the development of myopia, but the biological link between systemic processes and ocular changes remains poorly understood. In this study, we explored the gut–retina axis using a multi-omics approach in patients with high myopic cataract (HMC) compared with age-related cataract (ARC) controls. Fecal 16S rDNA sequencing revealed distinct microbial signatures in HMC, notably the enrichment of Lactobacillales and depletion of *Roseburia*, taxa associated with systemic inflammatory status. Metabolomic profiling of plasma and aqueous humor (AH) identified widespread alterations, with enrichment analyses highlighting pathways related to steroid hormone biosynthesis, PPAR signaling, and amino acid metabolism. Several metabolites correlated with axial length, and 4-phenolsulfonic acid in plasma and 5′-N-ethylcarboxamidoadenosine in AH showed the strongest discriminatory potential between HMC and ARC groups. While these findings are associative and require validation in larger, longitudinal cohorts, they suggest that systemic and ocular metabolic disturbances are closely linked to established high myopia. This study provides novel insights into the potential role of the gut–retina axis in myopia pathogenesis.

**Supplementary Information:**

The online version contains supplementary material available at 10.1038/s41598-025-23265-8.

## Introduction

Myopia is a common cause of vision loss. A total of 938 million people will have high myopia by 2050^1^. The prevalence of myopia in Eastern and Southeast Asia reached up to 80–90%^2^. High myopia that progressed from myopia brought a great burden on physical and mental health. Complications of high myopia such as myopic maculopathy, glaucoma, and early-onset cataracts deteriorate vision dramatically^3–5^. These comorbidities substantially reduce visual function, impose socioeconomic burdens, and diminish health-related quality of life in affected children and their families^6^. Accordingly, it is imperative to identify systemic and ocular biomarkers that reflect high myopia status and provide mechanistic insights into disease progression.

Several studies indicated a correlation between myopia and inflammation. Upregulated expression of c-Fos, nuclear factor κB (NF-κB), interleukin (IL)-6, and tumor necrosis factor (TNF)-α were demonstrated from an experimental myopia model^7^. Besides, in aqueous humor of high myopic patients, the levels of IL-1β, matrix metalloproteinase (MMP)-2, and IL-6 were significantly higher than in patients without high myopia, and concentrations of IL-6 and MMP2 were increased with axial lengths^8^. Upregulated IL-6 activated the TGF-β1/Smad2/MMP-2 pathway and promoted transdifferentiation of human scleral fibroblasts, resulting in sclera thinning and myopia progression^9^. Epidemiologic evidence further supports this link: patients with systemic inflammatory diseases such as type 1 diabetes, uveitis, and systemic lupus erythematosus exhibit higher prevalence of myopia^7^, and systemic inflammatory indices, including neutrophil-to-lymphocyte and platelet-to-lymphocyte ratios, are significantly elevated in high myopia^10^. However, the way systemic inflammation affects intraocular inflammation is still unknown.

To clarify this issue, we focused on the “Gut-retina axis”. Rowan S demonstrated the correlation between dietary patterns and age-related macular degeneration (AMD) via the gut–retina axis^11^. Afterward, gut microbial dysbiosis was proven to participate in ocular disorders that were associated with myopia such as uveitis, glaucoma, and dry eye disease (DED)^12–14^. In patients with uveitis, several anti-inflammatory genera were reduced, whereas a pro-inflammatory genus was enriched^12^. Glaucoma patients exhibit depletion of butyrate-producing bacteria^13^. And in dry eye disease (DED), shifts in phyla and genera correlate with disease severity^14^. Given these precedents, we hypothesized that alterations in gut microbiota may interact with systemic and ocular metabolic pathways to contribute to high myopia.

To test this hypothesis, we performed an integrated, cross-sectional multi-omics study analyzing gut microbiota, plasma metabolites, and aqueous humor metabolites in high myopic cataract (HMC) patients compared with age-related cataract (ARC) controls. By correlating microbial taxa and metabolites with axial length, we sought to uncover systemic and ocular signatures associated with high myopia. While the cross-sectional design precludes causal inference, our approach provides novel associative evidence for microbiome–metabolome–ocular interactions, thereby advancing understanding of the gut–eye axis in myopia.

## Results

### Patients characteristics

As Table 1 shows, we measured 54 feces specimens, 33 plasma specimens, and 33 aqueous humor specimens in this study after removing unqualified specimens. HMC patients involved in feces specimen analysis were younger (57.72 ± 8.27 years vs. 63.39 ± 11.26 years, *P* = 0.044). The AL and ACD of these patients were larger (AL: right eyes: 29.94 ± 2.52 mm vs. 23.57 ± 0.69 mm, *P* < 0.001; left eyes: 29.71 ± 1.64 mm vs. 23.79 ± 1.64 mm, *P* < 0.001; ACD: right eyes: 3.43 ± 0.47 mm vs. 3.04 ± 0.43 mm, *P* = 0.004; left eyes: 3.41 ± 0.38 mm vs. 3.05 ± 0.42 mm, *P* = 0.002) in both eyes. ARC and HMC patients have similar LT and WTW. In the plasma/AH cohort, groups were age-matched, though AL and ACD remained significantly higher in HMC patients.

**Table 1:** Characteristics of enrolled participants.

Characteristic	Feces specimens			Plasma and aqueous humor specimens		
	ARC	HMC	*P* value	ARC	HMC	*P* value
Patients, n	28	26	-	18 ^*^	15 ^*^	-
Female, n (%)	19(67.86)	22(84.61)	-	29(59.18) ^*^	25(75.76) ^*^	-
Age (yrs), Mean (SD)	63.39(11.26)	57.72(8.27)	0.044	64.67(12.04) ^*^	58.80(7.04) ^*^	0.107
AL (mm), Mean (SD)						
Right eye	23.57(0.69)	29.94(2.52)	< 0.001	23.72(0.70) ^*^	29.84(2.31) ^*^	< 0.001
Left eye	23.79(1.64)	29.71(2.73)	< 0.001	24.07(1.92)	29.14(2.61)	< 0.001
ACD (mm), Mean (SD)						
Right eye	3.04(0.43)	3.43(0.47)	0.004	3.15(0.42) ^*^	3.57(0.52) ^*^	0.017
Left eye	3.05(0.42)	3.41(0.38)	0.002	3.12(0.38)	3.46(0.33)	0.011
LT (mm), Mean (SD)						
Right eye	4.35(0.38)	4.08(0.84)	0.141	4.34(0.43) ^*^	4.01(1.06) ^*^	0.229
Left eye	4.27(0.43)	4.14(0.45)	0.311	4.29(0.49)	4.10(0.51)	0.300
WTW (mm), Mean (SD)						
Right eye	11.56(0.43)	11.72(0.41)	0.186	11.57(0.45) ^*^	11.71(0.40) ^*^	0.350
Left eye	11.54(0.39)	11.66(0.51)	0.353	11.60(0.42)	11.67(0.54)	0.699

AL = Axial length; ACD = Anterior chamber depth; LT = Lens thickness; WTW = White-to-white; SD = Standard Deviation.

Asterisk means the data included in the analysis of aqueous humor specimens.

### Profile of gut microbiome in high myopic cataract patients

**Fig. 1:**
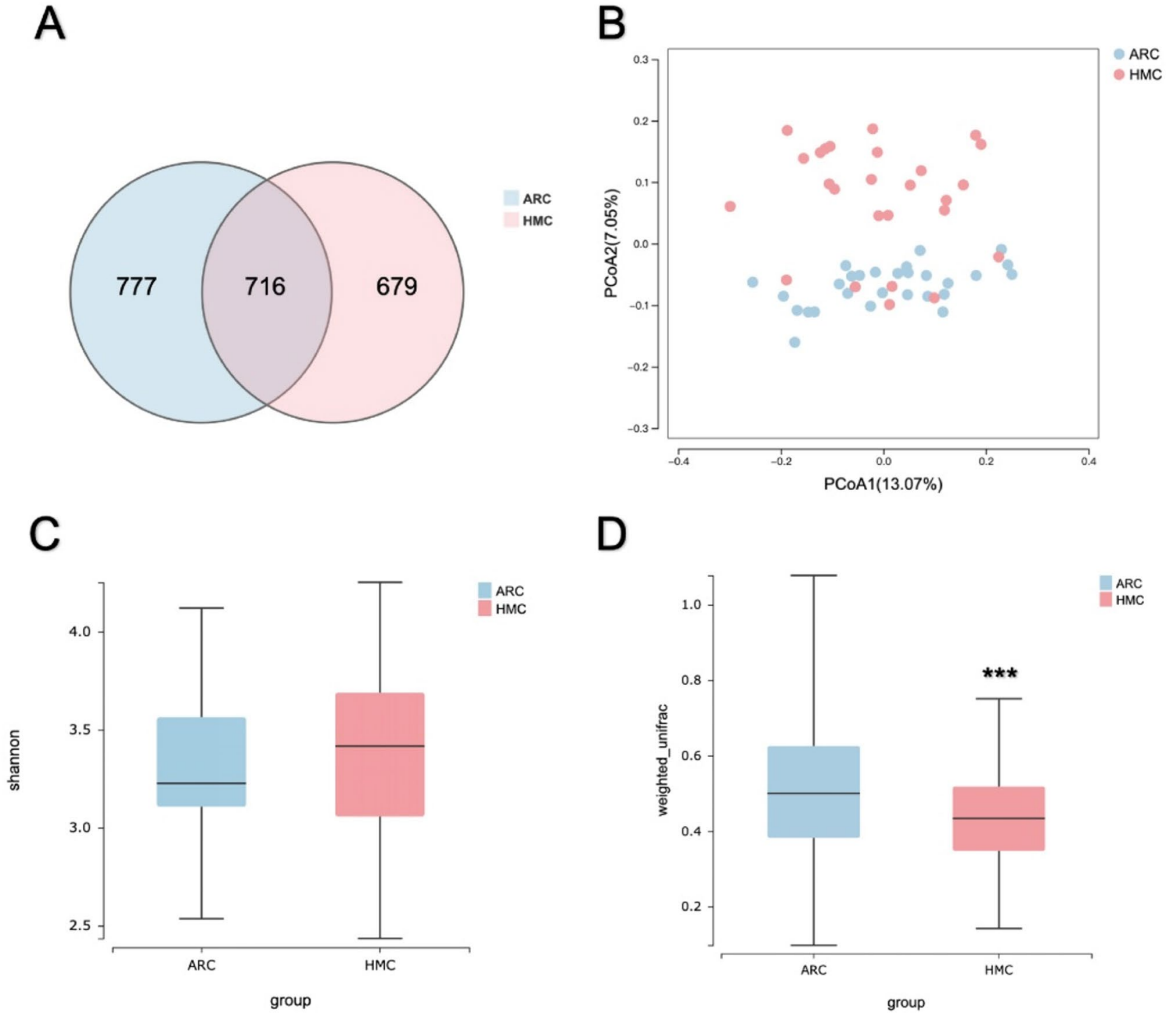
Gut microbiome diversity in ARC and HMC patients. (**A**) Venn diagram of the gut microbiome in age-related cataract patients (ARC) and high myopic cataract patients (HMC). (**B**) Principal coordinate analysis (PCoA) plot showed distinct clustering patterns of ARC and HMC groups. (**C**) Alpha-diversity with Shannon index showed similar intraindividual diversity of the gut microbiomes between two groups. (**D**) Significantly lower beta diversity was shown in HMC group. The “***” means “P < 0.001”.

As Fig. 1A shows, 1395 and 1493 OTUs were clustered in HMC and ARC groups respectively. Principal coordinate analysis (PCoA) was used to assess the variability of gut microbiome between two groups and found distinct clustering patterns of two groups (Fig. 1B). Figure 1C and D show intra- and interindividual diversity of the gut microbiomes. We found lower interindividual diversity of the gut microbiomes in the HMC group via beta diversity (*P* < 0.001).

**Fig. 2:**
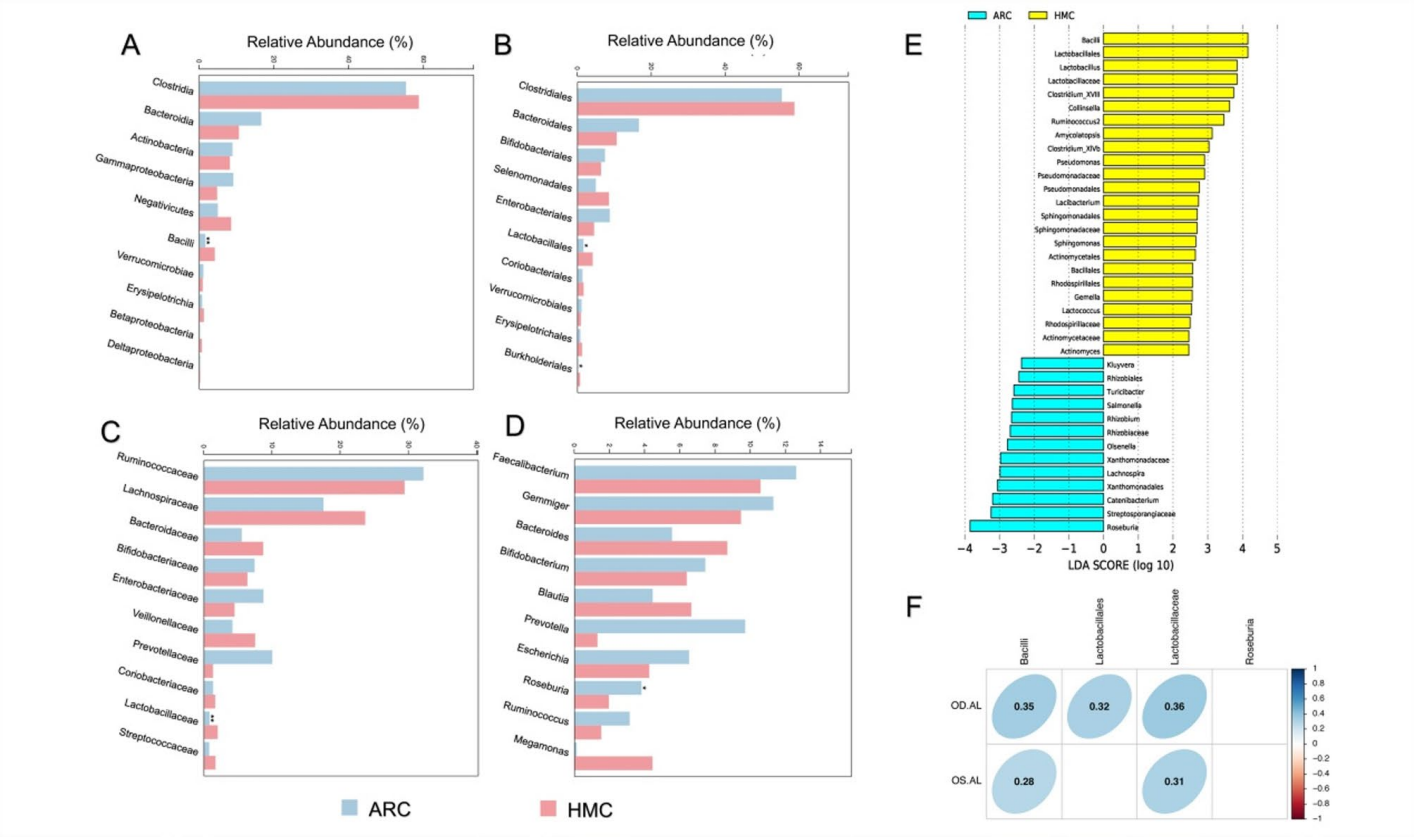
Differential microbial taxa between ARC and HMC patients. (**A**) The distinct gut taxa of HMC in levels of class was Bacilli, (**B**) in the level of order were Lactobacillales and Burkholderiales, (**C**) in the level of family was Lactobacillaceae, (**D**) in the level of genus was *Roseburia*. (**E**) LEfSe and linear discriminant analysis (LDA) scores results were shown. (**F**) Bacilli, Lactobacillaceae were associated with axial length (AL) in both eyes, whereas Lactobacillales was only associated with right AL.

Taxonomic profiling showed increased relative abundance of Bacilli (class) (4.16% vs. 1.64%, *P* = 0.008, Fig. 2A), Lactobacillales (order) (4.12% vs. 1.62%, *P* = 0.013, Fig. 2B), and Lactobacillaceae (family) (0.71% vs. 0.19%, *P* = 0.047, Fig. 2C) in HMC patients (all *P* < 0.05), alongside depletion of *Roseburia* (genus) (1.95% vs. 3.81%, *P* = 0.012, Fig. 2D). LEfSe identified 24 taxa enriched in HMC and 13 in ARC. Notably, Lactobacillus was strongly enriched in HMC patients (LDA = 3.84, *P* = 0.002; Table S1) (Fig. 2E). These findings suggest a shift towards pro-inflammatory gut taxa in high myopia. For example, Lactobacillaceae are associated with obesity and systemic inflammation (both obesity and inflammation have been linked to myopia risk), and *Roseburia* produces anti-inflammatory short-chain fatty acids (SCFA).

Figure 2F shows the results of Spearman correlation between distinct taxa and AL. We found Bacilli and Lactobacillaceae were positively correlated with AL in both eyes (Bacilli: right eye: *r* = 0.35, *P* = 0.012, left eye: *r* = 0.28, *P* = 0.043; Lactobacillaceae: right eye: *r* = 0.36, *P* = 0.009, left eye: *r* = 0.31, *P* = 0.027). In addition, Lactobacillales also positively correlated with right-eye AL (*r* = 0.32, *P* = 0.021). These findings suggest that enrichment of pro-inflammatory taxa and depletion of butyrate producers may contribute to a systemic milieu linked to axial elongation.

### Profile of plasma metabolites and aqueous humor metabolites in high myopic cataract patients

**Fig. 3:**
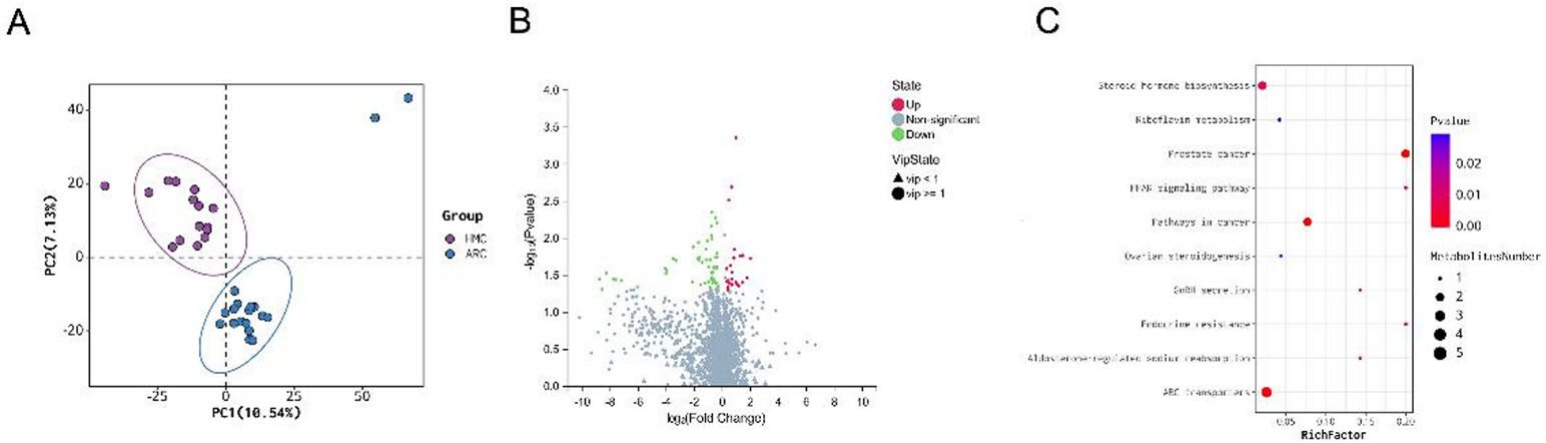
Plasma metabolomic differences between ARC and HMC groups. (**A**) PLS-DA model plot showed different clustering patterns in plasma metabolites of ARC and HMC groups. (**B**) The volcano plot showed 55 downregulated metabolites and 24 upregulated metabolites in the HMC group compared with the ARC group. (**C**) Bubble diagram shows the Top 10 enriched pathways through KEGG analysis^15–17^.

Plasma metabolomic profiling identified 79 significantly altered metabolites across chemical classes including amino acids, fatty acyls, and steroids (Fig. S2A, Table S2). After FDR correction, 14 metabolites remained statistically significant (FDR < 0.05, Table S2). Other additional metabolites showed suggestive associations (0.05 ≤ FDR < 0.10, Table S2). PLS-DA revealed clear group separation without evidence of model overfitting (R²Y = 0.996, Q²=0.017; Fig. 3A, S1A). Volcano analysis revealed 55 downregulated and 24 upregulated metabolites in HMC relative to ARC (Fig. 3B). KEGG pathway enrichment implicated ABC transporters, steroid hormone biosynthesis, and PPAR signaling (Fig. 3C). These systemic metabolic alterations are biologically plausible, given roles in retinal lipid homeostasis, scleral remodeling, and ocular growth regulation.

**Fig. 4:**
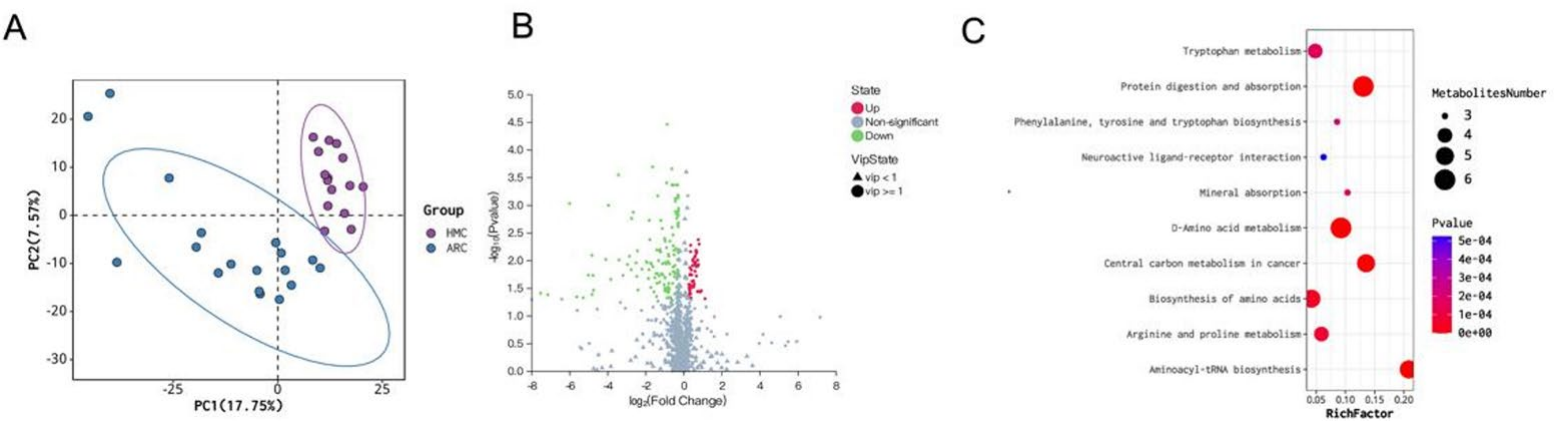
Aqueous humor (AH) metabolomic differences between ARC and HMC groups. (**A**) PLS-DA model plot showed different clustering patterns in aqueous humor (AH) metabolites of ARC and HMC groups. (**B**) The volcano plot showed 137 downregulated metabolites and 60 upregulated metabolites in the HMC group compared with the ARC group. (**C**) Bubble diagram of the top 10 pathways were shown^15–17^.

Aqueous humor metabolomics identified 197 significantly altered metabolites (60 upregulated, 137 downregulated) (Fig. 4B, Fig S2B, Table S4). They mainly consisted of amino acids (17), benzene and derivatives (17), organic acids (12), fatty acyls (10), amines (6), carbonyl compounds (6), and purines and derivatives (6). After FDR correction, 195 metabolites remained statistically significant (FDR < 0.05, Table S4), and other 2 metabolites showed suggestive associations (0.05 ≤ FDR < 0.10, Table S4). PLS-DA confirmed clear separation (R²Y = 0.988, Q²=0.389; Fig. 4A, S1B). KEGG analysis identified 44 significantly enriched pathways, of which the most relevant were protein digestion and absorption, aminoacyl-tRNA biosynthesis, D-amino acid metabolism, and biosynthesis of amino acids (Fig. 4C). All top pathways were predominantly downregulated, suggesting impaired amino acid metabolism within the ocular environment of high myopia. These integrated findings highlight systemic and local metabolic disruptions plausibly contributing to ocular elongation and scleral remodeling.

### The correlation between gut microbiome and plasma, aqueous humor metabolites

**Fig. 5:**
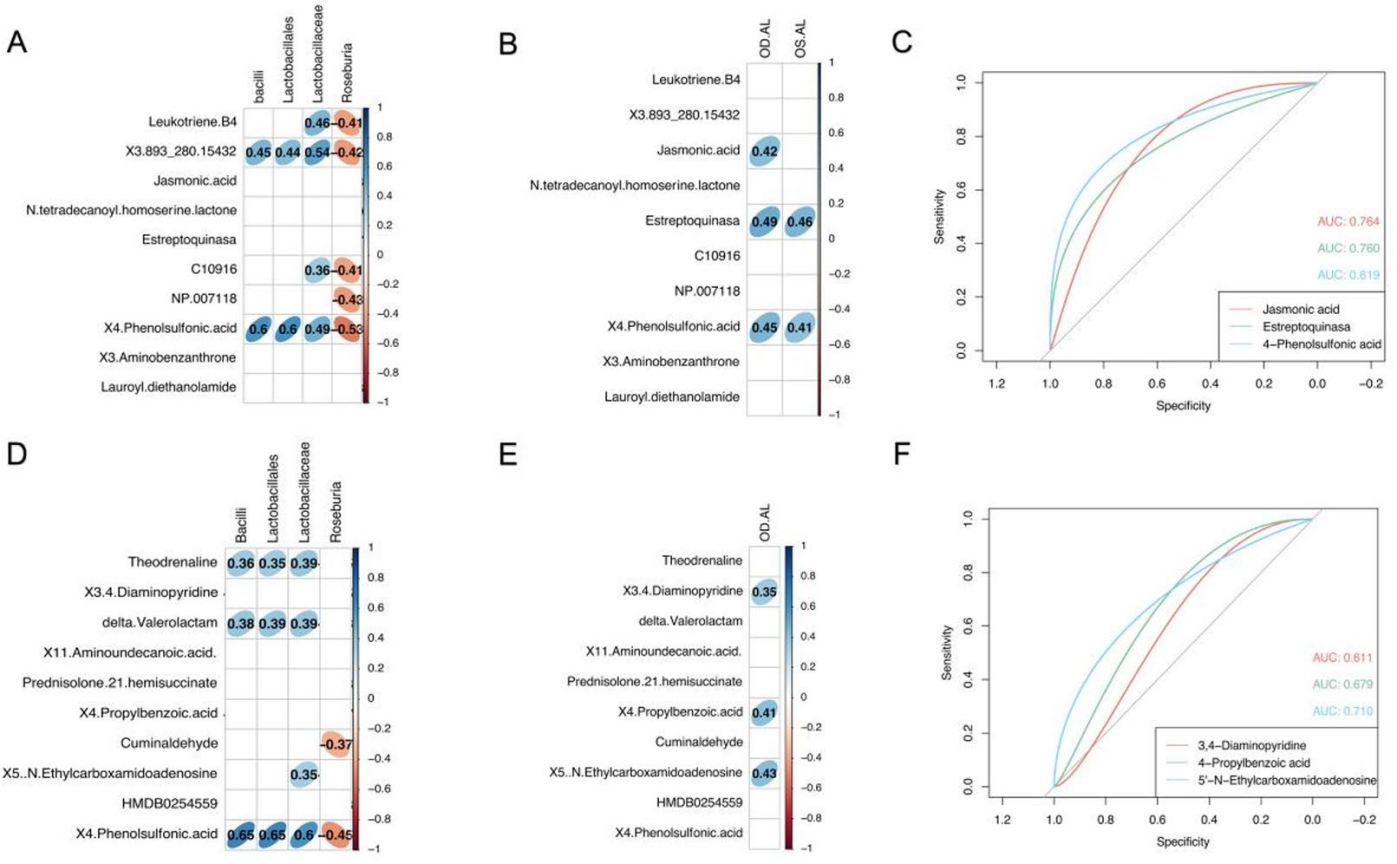
Correlation between gut microbiota, metabolites, and axial length. (**A**) The correlation between gut taxa and plasma metabolites. Only the results with P less than 0.05 were shown. The right side is the correlation coefficient, blue indicates positive correlation and red indicates negative correlation. (**B**) The correlation between the top 10 plasma metabolites and both eyes’ axial lengths. (**C**) The receiver operating characteristics (ROC) curve using axial lengths related plasma metabolites to predict the incidence of high myopia. (**D**) The correlation between gut taxa and aqueous humor (AH) metabolites. (**E**) The correlation between the top 10 AH metabolites and right eyes’ axial lengths. (**F**) The ROC curve using axial lengths related AH metabolites to predict the incidence of high myopia.

Correlation analyses linked gut microbiota with plasma and AH metabolites. Figure 5A shows the relationship between gut microbiome and plasma metabolites. We found Bacilli and Lactobacillales had positive correlations with (3aR,4aS,5R,8 S,9aR)-5-hydroxy-4a,8-dimethyl-3-methylidene- 2 H,3 H,3aH,4 H,4aH,5 H,6 H,8 H,9 H,9aH-azuleno[6,5-b]furan-2,6-dione (3.893_280.15432, *r* = 0.45, *P* = 0.008; *r* = 0.44, *P* = 0.011) and 4- Phenolsulfonic acid (*r* = 0.60, *P* < 0.001; *r* = 0.60, *P* < 0.001). Lactobacillaceae was positively correlated with leukotriene B4 (*r* = 0.46, *P* = 0.007), (3aR,4aS,5R,8 S,9aR)-5-hydroxy-4a,8-dimethyl-3-methylidene-2 H,3 H,3aH,4 H,4aH,5 H,6 H,8 H,9 H,9aH-azuleno[6,5-b]furan-2,6-dione(3.893_280.15432, *r* = 0.54, *P* = 0.001), 2-Dimethylamino-5,6-dimethyl pyrimidine-4-ol (C10916, *r* = 0.36, *P* = 0.041) and 4-Phenolsulfonic acid (*r* = 0.49, *P* = 0.004). *Roseburia* was negatively related with leukotriene B4 (*r*=-0.41, *P* = 0.017), (3aR,4aS,5R,8 S,9aR)-5-hydroxy- 4a,8-dimethyl-3-methylidene-2 H,3 H,3aH,4 H,4aH,5 H,6 H,8 H,9 H,9aH-azuleno[6,5-b]furan-2,6-dione (3.893_280.15432, *r*=-0.42, *P* = 0.013), 2-Dimethylamino-5,6-dimethyl pyrimidine-4-ol (C10916, *r*=-0.41, *P* = 0.018 ), NP.007118 (*r*=-0.43, *P* = 0.013) and 4-Phenolsulfonic acid (*r*=-0.53, *P* = 0.002).

Figure 5D shows the relationship between gut taxa and aqueous humor metabolites. Bacilli and Lactobacillales positively correlated with theodrenaline (*r* = 0.36, *P* < 0.001; *r* = 0.35, *P* < 0.001), delta-valerolactam (*r* = 0.38, *P* < 0.001; *r* = 0.39, *P* < 0.001) and 4-Phenolsulfonic acid (*r* = 0.65, *P* < 0.001; *r* = 0.65, *P* < 0.001). For Lactobacillaceae, theodrenaline (*r* = 0.39, *P* = 0.025), delta-valerolactam (*r* = 0.39, *P* = 0.027), 5’-N-Ethylcarboxamidoadenosine (*r* = 0.36, *P* < 0.001), and 4-Phenolsulfonic acid (*r* = 0.60, *P* = 0.049) were all positive correlated factors. Cumin aldehyde (*r*=-0.37, *P* = 0.036) and 4-Phenolsulfonic acid (*r*=-0.45, *P* = 0.008) were negative correlators of *Roseburia.*

### The relationship between plasma, aqueous humor metabolites and clinical parameters

Using Spearman correlation analysis, we explored the relationship between the top 10 elevated plasma metabolites and axial lengths in both eyes. We found jasmonic acid, estreptoquinasa and 4-Phenolsulfonic acid were positively correlated with axial lengths in the right eyes (Fig. 5B, *r* = 0.42, *P* = 0.014; *r* = 0.49, *P* = 0.003; *r* = 0.45, *P* = 0.008, respectively). As to left eye axial lengths, estreptoquinasa and 4-Phenolsulfonic acid were proved positively correlated (Fig. 5B, *r* = 0.46, *P* = 0.007; *r* = 0.41, *P* = 0.019, respectively). The receiver operating characteristic (ROC) curve based on the logistic regression model and the area under the curve (AUC) were conducted to select the most associated metabolite of high myopia. We evaluated the relationship between axial-length associated plasma metabolites and high myopia and found that 4-Phenolsulfonic acid had the best discriminatory capacity (Jasmonic acid: AUC = 0.764, 95%CI = 0.576–0.893; Estreptoquinasa: AUC = 0.760, 95%CI = 0.566–0.901; 4-Phenolsulfonic acid: AUC = 0.819, 95%CI = 0.642–0.936; Fig. 5C).

We used a similar procedure to analyze the relationship between aqueous humor metabolites and ocular parameters. We found 3,4-Diaminopyridine, 4-Propylbenzoic acid, and 5’-N-Ethylcarboxamidoadenosine were positively correlated with axial lengths in right eyes (Fig. 5E, *r* = 0.35, *P* = 0.045; *r* = 0.41, *P* = 0.018; *r* = 0.43, *P* = 0.013, respectively). Using the ROC curve and AUC, we identified 5’-N-Ethylcarboxamidoadenosine as the most associated metabolite with the best discriminatory capacity (3,4-Diaminopyridine: AUC = 0.611, 95%CI = 0.421–0.804; 4-Propylbenzoic acid: AUC = 0.679, 95%CI = 0.485–0.856; 5’-N-Ethylcarboxamidoadenosine: AUC = 0.710, 95%CI = 0.528–0.875; Fig. 5F).

## Discussion

Multiple studies have demonstrated correlations between systemic inflammation and ocular diseases via the “gut–retina axis”. For example, gut microbial communities are altered in patients with POAG, AMD, and DED^11,13,14^. There has been a longstanding hypothesis that myopia is associated with systemic inflammation, but direct evidence has been limited. Considering this gap, we conducted a cross-sectional multi-omics study to characterize gut microbiota, plasma, and aqueous humor (AH) metabolites in high myopic patients, aiming to determine whether the gut–retina axis might contribute to the pathogenesis of myopia. In this study, we found that high myopic cataract (HMC) patients differ significantly from age-related cataract (ARC) controls in gut microbiome composition as well as in systemic and ocular metabolite profiles. Although our findings are associative and do not establish causality, they strongly suggest that gut–retina axis alterations may be involved in high myopia.

We observed notable differences in the gut microbiota of HMC patients compared to ARC controls. Alpha diversity indices were similar between groups, consistent with a previous murine myopia model that found no significant change in alpha diversity^18^. Bacilli, Lactobacillales, and Lactobacillaceae were increased in HMC patients at the class, order, and family level. *Roseburia* was decreased in the HMC group at the genus level. When interpreting these differences, potential confounders such as age, diet, medication use, and systemic comorbidities must be considered. For instance, a shift toward a Bacteroidetes-dominated community has been observed in older individuals, but this alteration appears to be more closely linked to frailty rather than chronological age^19^. Moreover, lactic acid bacteria and Roseburia have been reported to remain relatively stable across the lifespan^20,21^. These observations suggest that the gut taxa differences identified in our study may be more strongly associated with systemic inflammation than with age itself. Dietary patterns also influence gut microbiota composition. For example, adherence to a Mediterranean diet has been associated with lower Escherichia coli counts and a higher Bifidobacteria: E. coli ratio, whereas a fast-food diet has been linked to reduced lactobacilli and butyrate-producing bacteria^22^. However, the patients in our cohort reported relatively homogeneous dietary habits, which may have minimized the potential confounding effect of diet. Medication use and systemic diseases were also considered, as commonly prescribed drugs—including proton pump inhibitors, metformin, antibiotics, and laxatives—are known to affect the gut microbiome^23^. Furthermore, alterations in gut microbial composition, such as reduced short-chain fatty acid–producing taxa and increased production of lipopolysaccharides and trimethylamine-N-oxide, have been implicated in the pathogenesis of geriatric diseases including diabetes, hypertension, and atherosclerosis^24,25^. To exclude these potential confounding factors, we conducted a detailed medical history inquiry for each patient.

The taxa-level changes we noted are consistent with earlier work, which helps account for the patterns observed. Family Lactobacillaceae is positively associated with obesity^26,27^, which is a proven related factor of myopia^28^. Obesity is accompanied by chronic systemic inflammation and leads to an increase of IL-6 and TNF-α in plasma^29,30^. These proinflammatory factors were also elevated in myopic aqueous humor^7,8^. In our HMC cohort, the enrichment of Lactobacillaceae correlated with longer axial lengths, further supporting a link between systemic inflammation and high myopia. Conversely, the lower abundance of *Roseburia* in HMC patients suggests a loss of anti-inflammatory short-chain fatty acid producers in myopia. *Roseburia* can produce short-chain fatty acids (SCFA, mainly butyrate) and then regulate intestinal physiology and immune homeostasis through anti-inflammatory properties^31^. Butyrate works in ameliorating uveitis via regulating Treg/Th17 in an autoimmune uveitis model^32^. Besides, in glaucoma patients, butyrate-producing taxa also decreased, and these taxa were proven protective associations with intraocular pressure (IOP) and optic nerve damage (vertical cup-to-disc ratio, VCDR)^13^. These indicate the proinflammatory environment that gut taxa cause can affect myopia. However, a small sample size study showed an enrichment of *Roseburia* in myopia patients^33^. We thought the diets of patients and sample size may lead to this difference because a previous study showed higher meat consumption increases the population size of *Roseburia*^34^.

Systemic metabolomic profiling revealed that HMC patients have a distinct plasma metabolite signature compared to controls. We identified 79 distinct plasma metabolites from 33 samples, which mainly belong to amino acids, benzene and derivatives, fatty acyls, organic acids, steroids and derivatives. Pathway analysis showed that these metabolites were enriched in pathways such as “ABC transporters,” “Steroid hormone biosynthesis,” and the “PPAR signaling pathway,” all of which have plausible links to ocular growth regulation. The serum metabolites of myopia in previous studies varied from 9 to 43, which are mainly amino acids, and organic acids^35–37^. Our broader panel of metabolites extends those findings and suggests additional pathways of interest. Several ABC transporters were co-localized at the blood-retina barrier (BRB) and were proven crucial in maintaining lipid homeostasis in the retinal pigment epithelium (RPE)^38^. Steroid hormones have also been implicated in eye growth: administering exogenous steroids was found to enhance axial elongation, induce myopic refractive shifts, and thin the sclera in a negative lens-induced myopia model in guinea pigs^39^. Similarly, the PPAR signaling pathway appears to play a role in modulating myopic progression. In a form-deprivation myopia model, peribulbar injections of PPARα agonist inhibited FDM progression, while the PPARα antagonist induced a myopic shift and downregulated the scleral COL1 expression^40^. Our detection of altered metabolites in the steroid hormone and PPAR pathways is partly consistent with these experimental observations, supporting the concept that systemic metabolic disturbances (particularly in lipid and energy metabolism) are associated with high myopia. Given that plasma metabolomics in high myopia has been rarely studied, these results provide new insights and broaden the perspective on the systemic aspects of myopia, warranting further investigation.

Within the eye, aqueous humor metabolomic analysis yielded even more pronounced differences between high myopia and controls. We found 60 upregulated and 137 downregulated metabolites in high myopia patients, including 17 amino acids, 17 benzene and derivatives, 12 organic acids,10 fatty acyls, 6 amines, 6 carbonyl compounds, and 6 purines and derivatives. Correspondingly, several amino acid-related pathways were enriched among the altered AH metabolites, including “Protein digestion and absorption,” “Aminoacyl-tRNA biosynthesis,” “D-amino acid metabolism,” and “Biosynthesis of amino acids.” These results are in line with prior studies that have characterized the ocular metabolome in myopia. Ji Y et al. reported a total of 242 metabolites from 40 AH samples (high myopia: control = 1:1), including 34 amino acids, 44 carbohydrates, 9 lipids, 7 nucleotides, other 148 metabolites, and 27 increased and 2 decreased metabolites were identified^41^. Another study compared samples from patients with normal axial length and with pathological myopia revealed 104 significantly different metabolites in myopic AH, including amino acids and purines^42^. Consistent with these reports, our study also found extensive changes in amino acid and purine metabolism in high myopia, and indeed we observed a greater number of metabolite differences, which may be attributable to our comprehensive profiling approach or differences in cohort characteristics. The more downregulated proteins were enriched in “Protein digestion and absorption” in this study. This finding resonates with proteomic analyses of diseased ocular environments; for example, in the AH of proliferative diabetic retinopathy, important structural proteins (including collagen types II, IX-α1, and IX-α2) that are relevant to myopia were downregulated and enriched in the same pathway^43^. In addition, D-Amino acid played an important role in neural development and was proven the coagonist of glutamate to activate Nmethyl-D-aspartate (NMDA) receptors^44^. The downregulation of “D-Amino acid metabolism” may affect the function of the retina, and be the accomplice of reduced glutamate to cause elongate axial length^18^. A study found that lymphocyte antigen 96 (LY-96) mRNA expression was increased in myopia and LY96 was negatively associated with the “Aminoacyl-tRNA biosynthesis” pathway^45^. This supports our finding of downregulation in the aminoacyl-tRNA biosynthesis pathway and suggests that impaired protein synthesis or turnover could be involved in myopic scleral remodeling. We found that “Biosynthesis of amino acids” pathways were also enriched. The detailed function of this pathway may need further experiments to explore.

Finally, we connected gut taxa, metabolites, and axial length via correlation analysis. We found the main gut taxa associated with metabolites were Bacilli (positively), Lactobacillales (positively), and *Roseburia* (negatively). In plasma metabolites, jasmonic acid, estreptoquinasa and 4-Phenolsulfonic acid were associated with axial length, while AH metabolites, 3,4-Diaminopyridine, 4-Propylbenzoic acid, and 5’-N-Ethylcarboxamidoadenosine were axial length associations. Of the above distinct metabolites, 4-Phenolsulfonic acid in plasma and 5’-N-Ethylcarboxamidoadenosine in AH were the most axial lengths-associated metabolite. These two metabolites demonstrated good discriminative ability between high myopic and control eyes in our data, suggesting they could serve as potential biomarkers for high myopia, pending validation in larger cohorts. The correlations identified here form a tentative network linking gut-derived and systemic metabolites to ocular changes, thereby providing a more holistic view of the gut–retina axis in myopia.

This study has several limitations that should be acknowledged. First, the cross-sectional design inherently precludes causal inference. Moreover, the temporal gap between the onset of high myopia (typically in adolescence) and cataract surgery (usually in middle to late adulthood) further complicates interpretation of directionality. Thus, it remains unclear whether alterations in the gut microbiome and metabolome contribute to the pathogenesis of high myopia, or conversely, whether high myopia and its associated lifestyle or dietary factors drive these changes. Longitudinal studies, ideally involving younger patients and tracking myopic progression over time, will be necessary to clarify the temporal dynamics of gut taxa and metabolite alterations relative to myopia onset. Second, potential confounders such as age, diet, and the presence of cataract may have influenced our findings. Although we used patients with age-related cataract as controls to approximate age matching, subtle differences in age distribution or systemic health status between groups could have contributed to variations in gut microbiota composition and metabolic profiles. Future studies employing more rigorously matched controls—preferably including healthy non-cataract individuals—and detailed dietary assessments are warranted to better isolate the effects of high myopia. Third, although many of the pathways identified in this study are supported by prior evidence, functional validation remains limited. Further mechanistic experiments are needed to elucidate the precise biological roles of these pathways in the context of high myopia. Finally, the relatively modest sample size may restrict both the statistical power and generalizability of our findings. Larger multicenter studies are required to confirm and strengthen the associations observed in the present analysis.

In conclusion, we identified significant shifts in gut microbiota composition, plasma metabolites, and aqueous humor (AH) metabolites in individuals with high myopia. Alterations in plasma and AH metabolites were associated with gut taxa and axial length (AL). Among them, plasma 4-phenolsulfonic acid and AH 5′-N-ethylcarboxamidoadenosine exhibited promising discriminatory potential. Although the study was performed in patients with established high myopia, these results provide novel insights into disease pathogenesis.

## Methods

### Study population and ophthalmic examinations

**Fig. 6:**
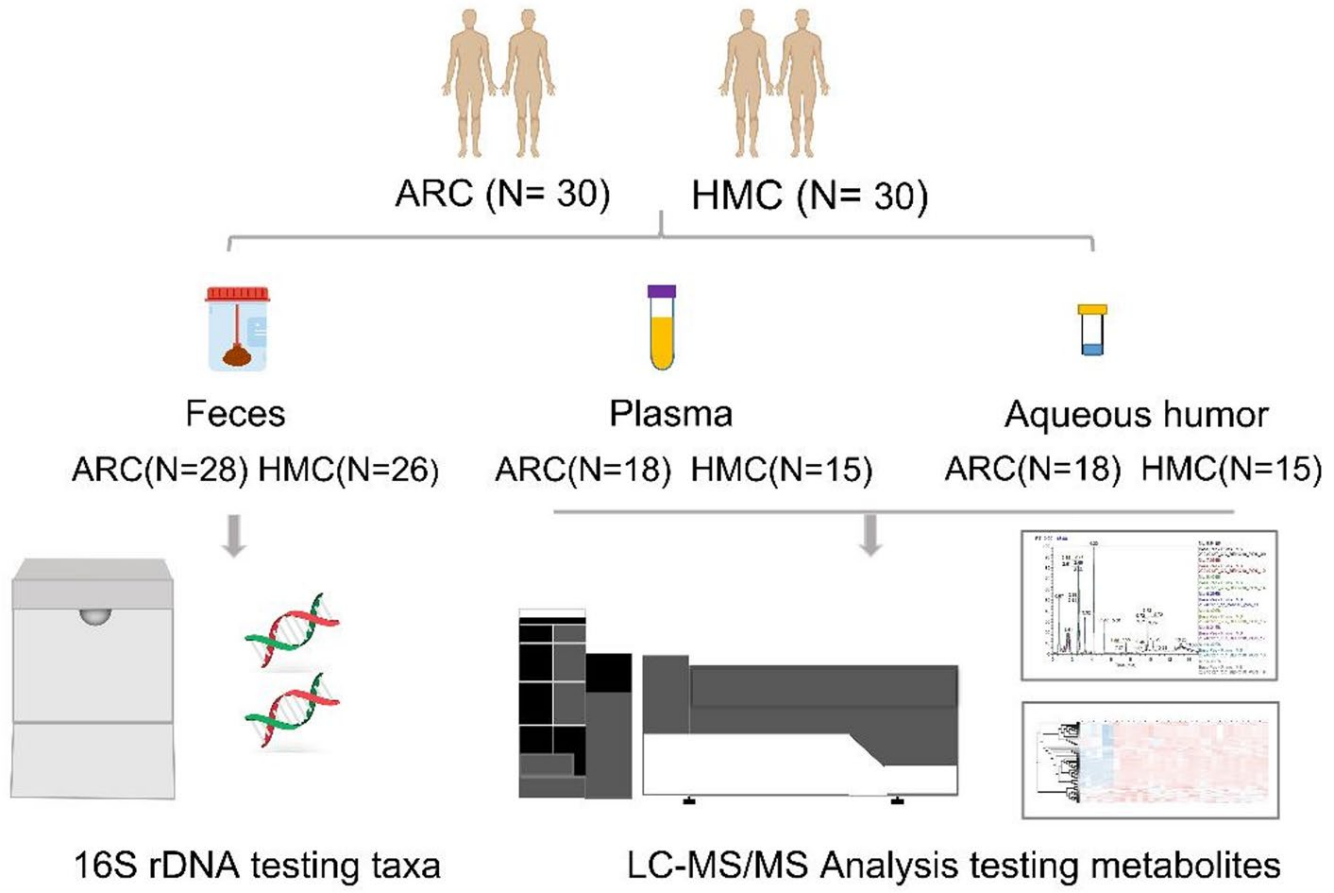
The flow chart of specimen collection and measurements in this study.

This study was conducted in Xi’an People’s Hospital (Xi’an Fourth Hospital) and was approved by the Xi’an People’s Hospital (Xi’an Fourth Hospital) Institutional Review Board (No.20230026). This single-center, cross-sectional study was performed in compliance with the tenets of the Declaration of Helsinki. Before the study, written informed consents were obtained from all the patients. The procedure of this study is shown in Fig. 6.

From January 2023 to July 2023, 60 patients who planned to undergo cataract surgery were included in this study. All patients were enrolled in Xi’an and were characterized by relatively homogeneous dietary habits, which helped to minimize potential confounding from dietary variation. Excluded criteria were as follows: (1) age was less than 40 years; (2) with a history of hypertension, diabetes and/or other systemic disease; (3) lens subluxation, glaucoma, ocular trauma, corneal scar, uveitis, and retinitis pigmentosa; (4) previous ocular surgery history, (5) taking anti-inflammatory agents, probiotics, or other medications that may influence gut microbiota.

After comprehensive ophthalmic examinations, all the patients finished IOL Master 700 (Carl Zeiss Meditec AG) measurements. Axial length (AL), anterior chamber depth (ACD), lens thickness (LT), and white-to-white (WTW) diameter were recorded.

### Feces specimens, plasma specimens, and aqueous humor specimens collection

Fecal samples were self-collected in sterile containers, stored at 4 °C for less than 6 h, and then frozen at − 80 °C. Peripheral blood was drawn after overnight fasting; plasma was separated and stored at − 80 °C. During phacoemulsification surgery, aqueous humor (AH, 100–150 µL) was aspirated under aseptic conditions prior to incision hydration and immediately cryopreserved at − 80 °C.

### Analysis of fecal gut Microbiome

Microbial DNA was extracted from fecal samples, and the V3–V4 region of 16 S rDNA was amplified for sequencing. After quality control, reads were clustered into operational taxonomic units (OTUs) at 97% similarity and taxonomically assigned against curated databases. Alpha and beta diversity indices were calculated, and differential taxa were identified using standard pipelines. Distinct taxa were further evaluated by linear discriminant analysis effect size (LEfSe). Detailed descriptions of sequencing quality control, filtering thresholds, and software parameters are provided in the Supplementary Methods.

### Metabolomic analysis of plasma and aqueous humor

Untargeted metabolomics was performed on plasma and AH samples using ultra-performance liquid chromatography coupled with high-resolution mass spectrometry (UPLC–MS/MS). Metabolites were identified by matching to established databases, and quality control was achieved using pooled QC samples. To obtain distribution difference in metabolites of the two groups, partial least squares-discriminant analysis (PLS-DA) was conducted via k-fold cross-validation (k = 7, stratified), to verify model robustness and exclude overfitting, response permutation testing (*n* = 200) also carried out. Differential metabolites were then defined based on fold change, significance testing, and variable importance in projection (VIP) scores. Using the Kyoto Encyclopedia of Genes and Genomes (KEGG) database, the metabolic pathways were explored^15–17^. The 10 metabolic pathways with the smallest P value were shown in the bubble chart. Complete details of extraction protocols, chromatographic conditions, mass spectrometer settings, and database matching criteria are provided in the Supplementary Methods.

### Statistical analysis

Statistical analysis was performed using R studio (version 4.2.2) and StataSE15 (version 15.0, Stata Corp LP, TX, USA). All continuous variables were expressed as means ± standard deviations (SD). Categorical variables were counted as values and percentages. The Shapiro-Francia W’ test was used to confirm the normal distribution. Student’s t-tests were used to assess the difference in ocular parameters between age-related cataract patients (ARC) and high myopic cataract patients (HMC). A two-sided P-value < 0.05 was considered statistically significant. We also applied the Benjamini–Hochberg false discovery rate (FDR) procedure. Both raw p-values and FDR are reported. Associations with FDR < 0.05 were considered still statistically significant, while associations with 0.05 ≤ FDR < 0.10 were considered suggestive^46^.

Associations between distinct gut taxa, metabolites, and ocular parameters were evaluated by Spearman correlation. Receiver operating characteristic (ROC) curves with area under the curve (AUC) statistics were generated to assess discriminatory performance of candidate metabolites.

## Supplementary Information

Below is the link to the electronic supplementary material.

Supplementary Material 1

Supplementary Material 2

Supplementary Material 3

Supplementary Material 4

## Data Availability

The datasets analysed during the current study are available in the Sequence Read Archive (SRA) with the Accession Number PRJNA1130869.
