# Supplementary material for: Integrative multi-omics identifies coordinated alterations in the gut microbiome and in plasma and aqueous humor metabolomes in high myopic cataract
Source: Sci Rep. 2025 Nov 12;15:39636. doi: 10.1038/s41598-025-23265-8 (PMC12612057; doi:10.1038/s41598-025-23265-8)
Supplement: Supplementary file 1 — Supplementary Material 1 [file 41598_2025_23265_MOESM1_ESM.docx]

**Table S1. LDA of gut taxa in the** **high myopic cataract patients and** **age-related cataract patients.**

| **Gut Taxa** | **Groups** | **LDA** | **P value** |
| --- | --- | --- | --- |
| Bacteria.Firmicutes.Bacilli.Lactobacillales.Lactobacillaceae.Lactobacillus | HMC | 3.84 | 0.002 |
| Bacteria.Firmicutes.Bacilli.Lactobacillales.Lactobacillaceae | HMC | 3.84 | 0.002 |
| Bacteria.Firmicutes.Clostridia.Clostridiales.Lachnospiraceae.Ruminococcus2 | HMC | 3.46 | 0.003 |
| Bacteria.Firmicutes.Bacilli | HMC | 4.16 | 0.004 |
| Bacteria.Firmicutes.Bacilli.Lactobacillales | HMC | 4.15 | 0.006 |
| Bacteria.Actinobacteria.Actinobacteria.Actinomycetales.Pseudonocardiaceae.Amycolatopsis | HMC | 3.13 | 0.008 |
| Bacteria.Firmicutes.Bacilli.Lactobacillales.Streptococcaceae.Lactococcus | HMC | 2.53 | 0.009 |
| Bacteria.Firmicutes.Erysipelotrichia.Erysipelotrichales.Erysipelotrichaceae.Clostridium_XVIII | HMC | 3.75 | 0.011 |
| Bacteria.Firmicutes.Bacilli.Bacillales.Bacillales | HMC | 2.57 | 0.012 |
| Bacteria.Firmicutes.Bacilli.Bacillales.Bacillales.Gemella | HMC | 2.55 | 0.012 |
| Bacteria.Actinobacteria.Actinobacteria.Actinomycetales.Actinomycetaceae.Actinomyces | HMC | 2.45 | 0.015 |
| Bacteria.Actinobacteria.Actinobacteria.Actinomycetales.Actinomycetaceae | HMC | 2.45 | 0.015 |
| Bacteria.Proteobacteria.Alphaproteobacteria.Rhodospirillales.Rhodospirillaceae.Lacibacterium | HMC | 2.73 | 0.016 |
| Bacteria.Firmicutes.Clostridia.Clostridiales.Lachnospiraceae.Clostridium_XlVb | HMC | 3.04 | 0.019 |
| Bacteria.Actinobacteria.Actinobacteria.Actinomycetales | HMC | 2.64 | 0.025 |
| Bacteria.Proteobacteria.Alphaproteobacteria.Rhodospirillales | HMC | 2.56 | 0.028 |
| Bacteria.Proteobacteria.Alphaproteobacteria.Rhodospirillales.Rhodospirillaceae | HMC | 2.49 | 0.028 |
| Bacteria.Proteobacteria.Alphaproteobacteria.Sphingomonadales | HMC | 2.70 | 0.030 |
| Bacteria.Proteobacteria.Alphaproteobacteria.Sphingomonadales.Sphingomonadaceae | HMC | 2.69 | 0.046 |
| Bacteria.Proteobacteria.Alphaproteobacteria.Sphingomonadales.Sphingomonadaceae.Sphingomonas | HMC | 2.65 | 0.046 |
| Bacteria.Actinobacteria.Actinobacteria.Coriobacteriales.Coriobacteriaceae.Collinsella | HMC | 3.63 | 0.046 |
| Bacteria.Proteobacteria.Gammaproteobacteria.Pseudomonadales.Pseudomonadaceae.Pseudomonas | HMC | 2.90 | < 0.001 |
| Bacteria.Proteobacteria.Gammaproteobacteria.Pseudomonadales.Pseudomonadaceae | HMC | 2.90 | < 0.001 |
| Bacteria.Proteobacteria.Gammaproteobacteria.Pseudomonadales | HMC | 2.76 | < 0.001 |
| Bacteria.Firmicutes.Clostridia.Clostridiales.Lachnospiraceae.Lachnospira | ARC | 2.98 | 0.003 |
| Bacteria.Firmicutes.Erysipelotrichia.Erysipelotrichales.Erysipelotrichaceae.Turicibacter | ARC | 2.59 | 0.002 |
| Bacteria.Proteobacteria.Gammaproteobacteria.Xanthomonadales | ARC | 3.06 | 0.047 |
| Bacteria.Proteobacteria.Gammaproteobacteria.Enterobacteriales.Enterobacteriaceae.Salmonella | ARC | 2.63 | 0.030 |
| Bacteria.Actinobacteria.Actinobacteria.Coriobacteriales.Coriobacteriaceae.Olsenella | ARC | 2.77 | 0.032 |
| Bacteria.Proteobacteria.Alphaproteobacteria.Rhizobiales.Rhizobiaceae | ARC | 2.69 | < 0.001 |
| Bacteria.Proteobacteria.Gammaproteobacteria.Xanthomonadales.Xanthomonadaceae | ARC | 2.97 | 0.047 |
| Bacteria.Firmicutes.Clostridia.Clostridiales.Lachnospiraceae.Roseburia | ARC | 3.85 | 0.014 |
| Bacteria.Actinobacteria.Actinobacteria.Actinomycetales.Streptosporangiaceae | ARC | 3.24 | 0.047 |
| Bacteria.Proteobacteria.Alphaproteobacteria.Rhizobiales | ARC | 2.44 | 0.027 |
| Bacteria.Proteobacteria.Alphaproteobacteria.Rhizobiales.Rhizobiaceae.Rhizobium | ARC | 2.65 | < 0.001 |
| Bacteria.Proteobacteria.Gammaproteobacteria.Enterobacteriales.Enterobacteriaceae.Kluyvera | ARC | 2.36 | 0.025 |
| Bacteria.Firmicutes.Erysipelotrichia.Erysipelotrichales.Erysipelotrichaceae.Catenibacterium | ARC | 3.20 | 0.025 |

HMC= high myopic cataract; ARC= age-related cataract; LDA= linear discriminant analysis scores.

**Table S2. Distinct plasm metabolites of high myopic cataract patients.**

| **Metabolite ID** | **Name** | **Ion Mode** | **Ratio**  **(HMC**  **vs ARC)** | **P value**  **(HMC**  **vs ARC)** | **VIP**  **(HMC**  **vs ARC)** | **FDR** |
| --- | --- | --- | --- | --- | --- | --- |
| B100069 | Creatine | POS | 1.613 | 0.002 | 2.401 | 0.079 |
| B100362 | Cortisone | POS | 0.782 | 0.018 | 1.542 | 0.084 |
| B300310 | Phenylalanylphenylalanine | POS | 0.015 | 0.046 | 2.712 | 0.049 |
| B300664 | Valylleucine | POS | 0.567 | 0.020 | 2.246 | 0.061 |
| B300735 | Umbelliferone | POS | 1.464 | 0.041 | 1.431 | 0.050 |
| 3.782_189.06621 | 6-methyl-5-nitroquinoline | POS | 0.781 | 0.006 | 1.410 | 0.079 |
| 3.893_280.15432 | (3aR,4aS,5R,8S,9aR)-5-hydroxy-4a,8-dimethyl-3-methylidene-2H,3H,3aH,4H,4aH,5H,6H,8H,9H,9aH-azuleno[6,5-b]furan-2,6-dione | POS | 3.489 | 0.035 | 2.685 | 0.059 |
| 5.504_696.43731 | PEG n15 | POS | 0.003 | 0.050 | 2.901 | 0.051 |
| 5.743_243.08786 | 3-amino-2-phenyl-2H-pyrazolo[4,3-c]pyridine-4,6-diol | POS | 0.511 | 0.025 | 2.015 | 0.062 |
| 5.929_300.21681 | NP-016386 | POS | 0.588 | 0.007 | 2.001 | 0.079 |
| B300189 | Yamogenin | POS | 0.713 | 0.018 | 1.630 | 0.079 |
| B300612 | Riboflavin | POS | 0.378 | 0.021 | 2.509 | 0.055 |
| Reference-3164 | PEG n10 | POS | 0.005 | 0.036 | 2.990 | 0.059 |
| Reference-3165 | PEG n11 | POS | 0.003 | 0.030 | 3.177 | 0.055 |
| Reference-3391 | Tiglic acid | POS | 0.696 | 0.025 | 1.581 | 0.060 |
| Reference-457 | Jasmonic acid | POS | 2.798 | 0.017 | 2.459 | 0.084 |
| Reference-512 | N-Acetyl-L-leucine | POS | 1.809 | 0.019 | 2.193 | 0.068 |
| Reference-674 | Testosterone | POS | 0.319 | 0.020 | 2.996 | 0.059 |
| Reference-965 | Diosgenin | POS | 0.713 | 0.018 | 1.630 | 0.075 |
| 3.485_250.14388 | N1-(2,3-dihydro-1,4-benzodioxin-2-ylmethyl)-2,2-dimethylpropanamide | POS | 0.316 | 0.012 | 3.060 | 0.086 |
| 3.939_409.18115 | NP-016596 | POS | 0.440 | 0.041 | 1.620 | 0.049 |
| 4.642_446.25938 | 1-(4-Methyl-1-piperazinyl)-2-[(3R,4S)-3-({5-[(phenylsulfanyl)methyl]-1,2-oxazol-3-yl}methyl)-4-piperidinyl]ethanone | POS | 0.005 | 0.037 | 2.987 | 0.054 |
| 4.982_318.18096 | (3R,4R)-N-Ethyl-4-hydroxy-3-[(4-methoxybenzoyl)amino]-1-azepanecarboxamide | POS | 0.725 | 0.005 | 1.613 | 0.079 |
| 8.165_363.29915 | 3-pentadecyl-4,5,6,7-tetrahydrobenzo[d]isoxazol-4-one oxime | POS | 1.394 | 0.003 | 1.878 | 0.079 |
| B300665 | N-acetylhomocitrulline | POS | 0.234 | 0.040 | 1.966 | 0.053 |
| Reference-308 | Alanyltyrosine | POS | 0.595 | 0.009 | 1.763 | 0.089 |
| Reference-6580 | Ethyl 3-hydroxybutyrate | POS | 0.065 | 0.028 | 2.556 | 0.057 |
| Reference-6771 | (R)-3-Hydroxy myristic acid | POS | 1.293 | 0.039 | 1.272 | 0.053 |
| Reference-8860 | (1R,9S)-11-(3-Methoxypropanoyl)-3-(2-thienyl)-7,11-diazatricyclo[7.3.1.02,7]trideca-2,4-dien-6-one | POS | 0.662 | 0.025 | 1.439 | 0.058 |
| C10916 | 2-Dimethylamino-5,6-dimethylpyrimidin-4-ol; 2-(Dimethylamino)-5,6-dimethyl-1H-pyrimidin-4-one; | POS | 2.314 | 0.045 | 2.254 | 0.049 |
| C17996 | Istamycin C1; | POS | 0.007 | 0.038 | 2.863 | 0.053 |
| C20128 | Bombykal; (Z,E)-10,12-Hexadecadienal; | POS | 0.785 | 0.025 | 1.495 | 0.056 |
| HMDB0000957 | Pyrocatechol | POS | 1.500 | 0.024 | 2.006 | 0.061 |
| HMDB0014883 | Modafinil | POS | 0.645 | 0.036 | 1.930 | 0.058 |
| HMDB0041679 | 4-Hydroxy-(3',4'-dihydroxyphenyl)-valeric acid | POS | 0.060 | 0.026 | 2.775 | 0.057 |
| HMDB0240660 | 5-Bromotryptophan | POS | 0.764 | 0.040 | 1.375 | 0.052 |
| HMDB0243905 | 1-Hydroxyvitamin D5 | POS | 1.366 | 0.037 | 1.449 | 0.053 |
| HMDB0243920 | 1-Methyl-1,2,3,4-tetrahydroisoquinoline | POS | 0.608 | 0.036 | 1.385 | 0.057 |
| HMDB0245815 | 3-Aminobenzanthrone | POS | 1.985 | 0.040 | 2.110 | 0.051 |
| HMDB0246912 | 1,2,4-Trimethoxy-5-propenylbenzene | POS | 1.654 | 0.042 | 1.740 | 0.049 |
| HMDB0248237 | alpha-Muramic acid | POS | 0.596 | 0.034 | 2.096 | 0.058 |
| HMDB0253333 | Ibufenac | POS | 1.336 | 0.050 | 1.364 | 0.050 |
| HMDB0254439 | Menoctone | POS | 1.617 | 0.043 | 1.952 | 0.049 |
| HMDB0256501 | Phthalic anhydride | POS | 1.357 | 0.046 | 1.408 | 0.048 |
| HMDB0256662 | Ethyl 2-(5-(4-chlorophenyl)pentyl)oxiran-2-carboxylate | POS | 0.609 | 0.010 | 2.307 | 0.088 |
| HMDB0256927 | Puromycin aminonucleoside | POS | 0.804 | 0.047 | 1.207 | 0.048 |
| HMDB0258508 | Estreptoquinasa | POS | 2.481 | 0.018 | 2.200 | 0.071 |
| HMDB0258594 | Sulfolane | POS | 0.349 | 0.042 | 2.342 | 0.048 |
| HMDB0260420 | N-[2-(5-Methoxy-1H-indol-3-yl)ethyl]-N-nitrosoacetamide | POS | 0.320 | 0.018 | 2.276 | 0.068 |
| LMFA07040039 | 12,13-Dimethyl-5,14-dioxabicyclo[9.2.1]-tetradeca-1(13),11-dien-4-one | POS | 1.639 | 0.029 | 1.842 | 0.057 |
| LMFA08030038 | N-tetradecanoyl-homoserine lactone | POS | 2.753 | 0.040 | 2.286 | 0.050 |
| LMFA08040058 | Lauroyl diethanolamide | POS | 1.834 | 0.014 | 2.272 | 0.085 |
| LMGP03060030 | 1-(2-methoxy-5Z,9Z-hexacosadienyl)-sn-glycero-3-phosphoserine | POS | 1.262 | 0.026 | 1.385 | 0.056 |
| LMPR0104010040 | Phytyl 2-O-methyl-dimethylarsinoylribose | POS | 0.002 | 0.036 | 3.187 | 0.056 |
| B100124 | D-Ribose | NEG | 0.613 | 0.004 | 2.333 | 0.079 |
| B200016 | 4-Vinylphenol sulfate | NEG | 0.608 | 0.036 | 1.841 | 0.055 |
| B200055 | Capryloylglycine | NEG | 0.615 | 0.030 | 1.902 | 0.054 |
| B300225 | Monobutyl phthalate | NEG | 1.606 | 0.036 | 2.006 | 0.054 |
| B300318 | Leukotriene B4 | NEG | 4.098 | 0.019 | 2.750 | 0.065 |
| Reference-1388 | Phenylacetaldehyde | NEG | 0.626 | 0.040 | 1.782 | 0.049 |
| Reference-1440 | 2,5-di-tert-Butylhydroquinone | NEG | 0.607 | 0.029 | 1.957 | 0.056 |
| Reference-550 | 4-Phenolsulfonic acid | NEG | 1.996 | 0.000 | 2.829 | 0.035 |
| 0.713_381.03895 | NP-007118 | NEG | 2.105 | 0.043 | 1.976 | 0.048 |
| 3.169_289.03854 | 3-(3-nitrophenyl)-2-phenylacrylic acid | NEG | 0.090 | 0.019 | 3.304 | 0.063 |
| 6.563_405.19183 | 20beta-Dihydroprednisone | NEG | 0.819 | 0.045 | 1.313 | 0.049 |
| 6.661_359.11696 | 2-[(4-Cyclopropyl-5-hydroxy-4H-1,2,4-triazol-3-yl)sulfanyl]-1-(4-morpholinophenyl)-1-ethanone | NEG | 0.310 | 0.019 | 3.083 | 0.060 |
| 7.411_293.17554 | NP-006255 | NEG | 0.631 | 0.029 | 1.819 | 0.055 |
| B100042 | L-Cystine | NEG | 0.550 | 0.046 | 1.613 | 0.048 |
| B300819 | D-chiro-Inositol | NEG | 0.711 | 0.041 | 1.729 | 0.048 |
| Reference-2560 | N-Acetyl-D-galactosamine | NEG | 0.469 | 0.020 | 2.315 | 0.056 |
| Reference-8256 | 7-Hydroxy-2-(4-hydroxyphenyl)-4-oxo-3,4-dihydro-2H-chromen-5-yl beta-D-glucopyranoside | NEG | 0.103 | 0.020 | 2.848 | 0.054 |
| C10412 | Uplandicine; 7-Acetyl-9-echimidinylretronecine; | NEG | 0.683 | 0.037 | 1.692 | 0.052 |
| HMDB0244558 | N-(p-Toluenesulfonyl)-L-phenylalanine | NEG | 0.480 | 0.011 | 2.316 | 0.087 |
| HMDB0245764 | Isoindole | NEG | 0.791 | 0.026 | 1.223 | 0.054 |
| HMDB0253025 | Phenylalanylleucine | NEG | 0.063 | 0.030 | 2.426 | 0.053 |
| HMDB0254858 | 1,3-Dihydroxypropan-2-yl formate | NEG | 0.787 | 0.016 | 1.541 | 0.084 |
| HMDB0257777 | [(2R,3S,4R,5R)-3,4,5,6-Tetrahydroxy-1-oxohexan-2-yl] (2S)-2-aminopropanoate | NEG | 0.457 | 0.014 | 2.778 | 0.079 |
| HMDB0258928 | Tetraphenylcyclopentadienone | NEG | 0.243 | 0.013 | 2.663 | 0.086 |
| HMDB0258932 | Tetrasul sulfoxide | NEG | 0.521 | 0.039 | 2.026 | 0.052 |

HMC= high myopic cataract; ARC= age-related cataract; VIP= Variable importance in the projection.

**Table S3. Pathways that were identified by KEGG analysis based on plasm metabolites of high myopic cataract patients.**

| **Pathway** | **Count** | **P value** | **FDR** | **KEGG Names** |
| --- | --- | --- | --- | --- |
| Prostate cancer | 2 | < 0.001 | 0.005 | Cortisone;Testosterone |
| ABC transporters | 3 | < 0.001 | 0.015 | D-Ribose;Riboflavin;L-Cystine |
| Pathways in cancer | 2 | < 0.001 | 0.012 | Cortisone;Testosterone |
| Endocrine resistance | 1 | 0.006 | 0.113 | Testosterone |
| PPAR signaling pathway | 1 | 0.006 | 0.090 | Leukotriene B4 |
| Steroid hormone biosynthesis | 2 | 0.007 | 0.081 | Cortisone;Testosterone |
| Aldosterone-regulated sodium reabsorption | 1 | 0.009 | 0.090 | Cortisone |
| GnRH secretion | 1 | 0.009 | 0.079 | Testosterone |
| Ovarian steroidogenesis | 1 | 0.029 | 0.228 | Testosterone |
| Riboflavin metabolism | 1 | 0.029 | 0.214 | Riboflavin |
| Ferroptosis | 1 | 0.036 | 0.234 | L-Cystine |
| Vitamin digestion and absorption | 1 | 0.037 | 0.222 | Riboflavin |
| Inflammatory mediator regulation of TRP channels | 1 | 0.041 | 0.225 | Leukotriene B4 |
| Pentose phosphate pathway | 1 | 0.044 | 0.228 | D-Ribose |
| Inositol phosphate metabolism | 1 | 0.047 | 0.224 | D-chiro-Inositol |
| Serotonergic synapse | 1 | 0.050 | 0.226 | Leukotriene B4 |
| alpha-Linolenic acid metabolism | 1 | 0.053 | 0.223 | Jasmonic acid |
| Glycine, serine and threonine metabolism | 1 | 0.054 | 0.215 | Creatine |
| Galactose metabolism | 1 | 0.055 | 0.209 | N-Acetyl-D-galactosamine |
| Protein digestion and absorption | 1 | 0.056 | 0.203 | L-Cystine |
| Neuroactive ligand-receptor interaction | 1 | 0.059 | 0.201 | Leukotriene B4 |
| Phenylalanine metabolism | 1 | 0.059 | 0.196 | Phenylacetaldehyde |
| Cysteine and methionine metabolism | 1 | 0.079 | 0.246 | L-Cystine |
| Arginine and proline metabolism | 1 | 0.082 | 0.246 | Creatine |
| Arachidonic acid metabolism | 1 | 0.093 | 0.270 | Leukotriene B4 |
| Bile secretion | 1 | 0.111 | 0.306 | Leukotriene B4 |
| Amino sugar and nucleotide sugar metabolism | 1 | 0.129 | 0.345 | N-Acetyl-D-galactosamine |
| Biosynthesis of nucleotide sugars | 1 | 0.223 | 0.575 | N-Acetyl-D-galactosamine |
| Biosynthesis of cofactors | 1 | 0.304 | 0.755 | Riboflavin |

**Table S4. Distinct aqueous humor metabolites of high myopic cataract patients.**

| **Metabolite ID** | **Name** | **Ion Mode** | **Ratio**  **(HMC vs ARC)** | **P value (HMC vs ARC)** | **VIP**  **(HMC vs ARC)** | **FDR** |
| --- | --- | --- | --- | --- | --- | --- |
| B100064 | L-Phenylalanine | POS | 0.811 | 0.001 | 1.233 | 0.020 |
| B100092 | Hippuric acid | POS | 0.517 | 0.011 | 1.846 | 0.029 |
| B100165 | Caprylic acid | POS | 0.264 | 0.011 | 1.965 | 0.028 |
| B100291 | N-Acetyl-L-methionine | POS | 0.767 | 0.039 | 1.138 | 0.043 |
| B200076 | (+/-)-Tryptophan | POS | 0.805 | < 0.001 | 1.296 | 0.086 |
| B200104 | N-(3-acetamidopropyl)pyrrolidin-2-one | POS | 1.354 | 0.024 | 1.235 | 0.035 |
| B200119 | 4-Ethoxybenzaldehyde | POS | 0.434 | 0.007 | 1.730 | 0.028 |
| B300176 | Phytosphingosine | POS | 1.235 | 0.047 | 1.165 | 0.049 |
| B300247 | Indole | POS | 0.813 | 0.001 | 1.238 | 0.018 |
| B300260 | Hexanoylcarnitine | POS | 0.549 | 0.048 | 1.367 | 0.049 |
| B300656 | Cyclo(L-prolyl-L-valyl) | POS | 0.598 | 0.012 | 1.655 | 0.028 |
| 3.23_126.09153 | NP-021733 | POS | 1.376 | 0.012 | 1.309 | 0.028 |
| 3.421_238.10760 | 5-Methoxy methylone | POS | 1.258 | 0.018 | 1.103 | 0.031 |
| 3.456_250.14386 | N1-(2,3-dihydro-1,4-benzodioxin-2-ylmethyl)-2,2-dimethylpropanamide | POS | 0.304 | 0.031 | 2.180 | 0.039 |
| 4.911_212.09198 | NP-019445 | POS | 0.150 | 0.002 | 2.975 | 0.016 |
| 5.333_189.09143 | NP-013210 | POS | 0.569 | 0.010 | 1.891 | 0.029 |
| 5.589_299.14884 | NP-009265 | POS | 1.471 | 0.026 | 1.542 | 0.037 |
| 7.918_460.26931 | NP-015331 | POS | 1.544 | 0.011 | 2.807 | 0.028 |
| 8.297_331.18779 | ethyl 3-amino-1-[6-(4-methylpiperidino)pyridazin-3-yl]-1H-pyrazole-4-carboxylate | POS | 1.290 | 0.029 | 1.588 | 0.039 |
| 8.298_221.11740 | 1-(3-ethyl-2,4-dihydroxy-6-methoxyphenyl)butan-1-one | POS | 1.276 | 0.030 | 1.519 | 0.040 |
| 8.556_330.33657 | Arachidic Acid | POS | 1.457 | 0.028 | 1.747 | 0.038 |
| 8.848_181.08618 | NP-012802 | POS | 1.212 | 0.027 | 1.111 | 0.037 |
| 8.848_315.19295 | 4-hydroxy-6-[2-(2-methyl-1,2,4a,5,6,7,8,8a-octahydronaphthalen-1-yl)ethyl]oxan-2-one | POS | 1.239 | 0.030 | 1.208 | 0.040 |
| B100154 | Methylglutaric acid | POS | 1.312 | 0.032 | 1.244 | 0.040 |
| B300063 | Methyl palmitate | POS | 0.059 | 0.010 | 2.622 | 0.029 |
| B300138 | Indole-3-carboxylic acid | POS | 0.634 | 0.049 | 1.362 | 0.050 |
| Reference-1332 | Diethyl phosphate | POS | 0.182 | 0.020 | 2.643 | 0.031 |
| Reference-142 | 3,4-Dihydroxyphenylpropionic acid | POS | 1.280 | 0.030 | 1.564 | 0.039 |
| Reference-1723 | 4-Indolecarbaldehyde | POS | 0.828 | 0.002 | 1.148 | 0.015 |
| Reference-1783 | D-(+)-Proline | POS | 0.746 | 0.001 | 1.346 | 0.016 |
| Reference-1817 | Heptanophenone | POS | 0.036 | 0.008 | 3.079 | 0.026 |
| Reference-191 | 3-Aminophenol | POS | 0.566 | 0.030 | 1.747 | 0.039 |
| Reference-2036 | Valethamate | POS | 0.588 | 0.047 | 1.940 | 0.049 |
| Reference-2088 | Ethyl paraben | POS | 0.095 | 0.010 | 2.580 | 0.028 |
| Reference-220 | 3-Succinoylpyridine | POS | 0.624 | 0.047 | 1.381 | 0.048 |
| Reference-2620 | 4-Propylbenzoic acid | POS | 1.725 | 0.036 | 1.554 | 0.042 |
| Reference-2744 | Acetophenone | POS | 0.825 | 0.007 | 1.117 | 0.028 |
| Reference-2816 | N,N-Diethylethanolamine | POS | 0.093 | < 0.001 | 3.825 | 0.028 |
| Reference-2844 | 1,5-Naphthalenediamine | POS | 0.830 | 0.001 | 1.169 | 0.015 |
| Reference-2872 | Cyromazine | POS | 0.139 | 0.008 | 2.354 | 0.025 |
| Reference-2882 | Eugenol | POS | 1.258 | 0.014 | 1.159 | 0.028 |
| Reference-2933 | 6-Methylquinoline | POS | 0.828 | 0.002 | 1.148 | 0.015 |
| Reference-2988 | delta-Valerolactam | POS | 1.840 | 0.037 | 1.893 | 0.042 |
| Reference-3313 | 3-Acetyl-2,5-dimethylfuran | POS | 1.438 | 0.030 | 1.263 | 0.039 |
| Reference-3408 | N-Nitrosomethylethylamine (NMEA) | POS | 0.583 | 0.026 | 1.433 | 0.037 |
| Reference-359 | Cuminaldehyde | POS | 1.712 | 0.012 | 2.537 | 0.027 |
| Reference-3769 | 15-(tert-Butyl)-2,3,5,6,8,9,11,12-octahydro-1,4,7,10,13-benzopentaoxacyclopentadecine | POS | 1.295 | 0.030 | 1.244 | 0.039 |
| Reference-401 | N-Acetyldopamine | POS | 0.526 | 0.033 | 1.978 | 0.040 |
| Reference-486 | Methionine | POS | 0.654 | < 0.001 | 1.804 | 0.015 |
| Reference-4992 | 4-Amino-3-hydroxybenzoic acid | POS | 0.541 | 0.036 | 1.918 | 0.041 |
| Reference-6329 | MDA 2-amido analog | POS | 0.587 | 0.032 | 1.558 | 0.040 |
| Reference-633 | 6-Methoxyquinoline N-oxide | POS | 0.459 | 0.003 | 2.229 | 0.017 |
| Reference-6558 | 2,4-Dimethylbenzaldehyde | POS | 1.590 | 0.013 | 2.080 | 0.027 |
| Reference-7170 | Prednisolone | POS | 0.005 | 0.039 | 2.590 | 0.042 |
| Reference-7379 | (5xi,9xi,16xi)-17-Hydroxykauran-19-oic acid | POS | 1.267 | 0.028 | 1.306 | 0.038 |
| Reference-7417 | 4-Ethoxy ethylbenzoate | POS | 0.031 | 0.019 | 2.646 | 0.031 |
| Reference-7437 | Bis(4-ethylbenzylidene)sorbitol | POS | 1.577 | 0.013 | 2.892 | 0.027 |
| Reference-7737 | 4-[(6E)-3-Hydroxy-8,10-dimethyl-2-(methylamino)-6-dodecen-1-yl]phenol | POS | 0.340 | 0.037 | 2.578 | 0.042 |
| Reference-930 | Triethyl phosphate | POS | 0.186 | 0.007 | 3.177 | 0.027 |
| Reference-939 | 1-Stearoylglycerol | POS | 1.292 | 0.040 | 1.180 | 0.043 |
| 0.706_353.09285 | ethyl 5-[3-(dimethylamino)allanoyl]-2-phenyl-1,3-thiazole-4-carboxylate | POS | 1.487 | 0.009 | 1.902 | 0.026 |
| 3.035_302.19623 | NP-016437 | POS | 1.600 | 0.007 | 3.012 | 0.027 |
| 3.319_174.09166 | Pyroquilon | POS | 0.831 | 0.001 | 1.181 | 0.014 |
| 3.337_192.10547 | INH | POS | 0.819 | 0.001 | 1.208 | 0.013 |
| 3.339_336.19171 | (3R,4R)-N-Ethyl-4-hydroxy-3-[(4-methoxybenzoyl)amino]-1-azepanecarboxamide | POS | 0.687 | 0.013 | 1.475 | 0.026 |
| 3.79_269.06250 | 3-Methyl-5-(5-methylisoxazol-3-yl)-4-(5-methyl-1,2,4-oxadiazol-3-yl)isoxazole | POS | 0.286 | 0.002 | 2.892 | 0.014 |
| 4.625_194.08156 | methyl 2-(benzoylamino)acetate | POS | 0.551 | < 0.001 | 2.148 | 0.002 |
| 5.058_333.18826 | N'-[4-(tert-butyl)phenyl]-N-cyclohexyl-N-methylurea | POS | 1.316 | 0.048 | 2.281 | 0.049 |
| 5.347_152.10726 | 2-((Dimethylamino)methyl)phenol | POS | 0.583 | 0.031 | 1.732 | 0.039 |
| 5.349_387.15512 | NP-002582 | POS | 0.278 | 0.021 | 2.803 | 0.032 |
| 5.439_247.04758 | 8-chloro-7-methyl-5-(2-pyridyl)[1,2,3,4]tetraazolo[1,5-c]pyrimidine | POS | 0.130 | 0.012 | 2.317 | 0.027 |
| 6.106_288.21683 | NP-002676 | POS | 0.437 | 0.002 | 2.260 | 0.014 |
| 7.266_199.13080 | HLK | POS | 0.352 | 0.021 | 2.010 | 0.032 |
| 7.576_280.04782 | O3-(3-chlorobenzoyl)-5-methylisoxazole-3-carbohydroximamide | POS | 0.570 | 0.033 | 1.464 | 0.040 |
| 7.577_294.06351 | 3-[1-(dimethylamino)-1H-pyrrol-2-yl]-2-(2-furylcarbonyl)acrylonitrile | POS | 0.500 | 0.004 | 1.872 | 0.021 |
| 7.916_517.13701 | (2R,3S,4S,5R,6S)-2-({[(2S,3R,4R)-3,4-dihydroxy-4-(hydroxymethyl)oxolan-2-yl]  oxy}methyl)-6-(3,4,5-trimethoxyphenoxy)oxane-3,4,5-triol | POS | 1.599 | 0.019 | 2.492 | 0.031 |
| 9.357_352.24570 | (1R,2S,3R,5S,6R,8aR)-5-[2-(furan-3-yl)ethyl]-5,6,8a-trimethyl-octahydro-2H-spiro  [naphthalene-1,2'-oxirane]-2,3-diol | POS | 1.392 | 0.010 | 1.729 | 0.028 |
| B100066 | L-Aspartic acid | POS | 0.744 | 0.001 | 1.443 | 0.012 |
| B300166 | Hexadecasphingosine (d16:1) | POS | 0.506 | 0.032 | 1.349 | 0.039 |
| Reference-1369 | Olomoucine | POS | 1.286 | 0.018 | 1.468 | 0.031 |
| Reference-1540 | L-5-Hydroxytryptophan | POS | 0.827 | 0.001 | 1.213 | 0.012 |
| Reference-1576 | Benzyladenine | POS | 0.444 | 0.017 | 2.123 | 0.030 |
| Reference-1765 | Tetramethylene sulfoxide | POS | 0.418 | 0.025 | 2.365 | 0.036 |
| Reference-215 | Skatole | POS | 0.826 | 0.002 | 1.153 | 0.013 |
| Reference-2728 | Cyclohexanecarboxylic acid | POS | 0.364 | 0.017 | 1.691 | 0.030 |
| Reference-2754 | Sorbic acid | POS | 1.298 | 0.005 | 1.216 | 0.023 |
| Reference-3070 | Propoxur | POS | 0.148 | 0.015 | 2.567 | 0.029 |
| Reference-3145 | Crotetamide | POS | 0.402 | 0.010 | 2.265 | 0.027 |
| Reference-584 | Atenolol | POS | 0.016 | 0.001 | 3.977 | 0.011 |
| Reference-6717 | 9-Nitrooleate | POS | 1.229 | 0.030 | 1.169 | 0.038 |
| Reference-695 | Dimethyl cyclohexane-1,4-dicarboxylate | POS | 0.327 | 0.013 | 1.946 | 0.026 |
| Reference-7533 | 1. (4-Carboxy-3-methylbutyl)-1,4a-dimethyl-6-methylenedecahydro 2. -1-naphthalenecarboxylic acid | POS | 1.299 | 0.028 | 1.647 | 0.038 |
| Reference-7607 | (8aR,12S,12aR)-12-Hydroxy-4-methyl-4,5,6,7,8,8a,12,12a-octahydro-2H  -3-benzoxecine-2,9(1H)-dione | POS | 1.232 | 0.041 | 1.491 | 0.044 |
| Reference-8649 | 6-Amino-3-methyl-1-phenyl-1H-pyrazolo[3,4-b]pyridine-5-carboxamide | POS | 0.506 | 0.035 | 2.071 | 0.041 |
| Reference-935 | Valerophenone | POS | 0.037 | 0.019 | 2.628 | 0.030 |
| C04126 | L-1-Aminopropan-2-ol O-phosphate; (S)-1-Aminopropan-2-yl phosphate | POS | 0.568 | 0.036 | 1.578 | 0.041 |
| C09570 | Tutin | POS | 1.508 | 0.012 | 2.787 | 0.027 |
| C14512 | Benzofuran | POS | 0.769 | 0.001 | 1.278 | 0.010 |
| C17783 | Capillanol | POS | 0.036 | 0.041 | 2.328 | 0.044 |
| C18380 | Calcium levulinate anhydrous | POS | 0.586 | 0.008 | 1.665 | 0.025 |
| C19127 | Proxan | POS | 0.494 | 0.027 | 2.065 | 0.037 |
| C19325 | 11-Aminoundecanoic acid | POS | 1.762 | 0.005 | 3.278 | 0.023 |
| C21414 | Naphthalene-1,2,4,8-tetrol | POS | 0.508 | 0.020 | 1.877 | 0.031 |
| HMDB0000063 | Cortisol | POS | 0.284 | 0.015 | 1.957 | 0.029 |
| HMDB0014883 | Modafinil | POS | 1.346 | 0.018 | 1.748 | 0.030 |
| HMDB0030961 | 2-Octenal | POS | 0.220 | 0.014 | 2.014 | 0.027 |
| HMDB0243886 | 1-Heptanesulfonic acid | POS | 0.378 | 0.026 | 1.856 | 0.037 |
| HMDB0243960 | 1-Naphthyl isocyanate | POS | 0.810 | 0.002 | 1.214 | 0.013 |
| HMDB0244016 | Vinylcyclohexene | POS | 0.178 | 0.011 | 2.169 | 0.027 |
| HMDB0244246 | 1,7-Octadiene | POS | 0.537 | 0.006 | 1.582 | 0.026 |
| HMDB0245245 | 2-n-Propylthiazolidine-4-carboxylic acid | POS | 0.376 | 0.045 | 2.160 | 0.047 |
| HMDB0245765 | 2H-Pyran-2-one | POS | 1.294 | 0.007 | 1.366 | 0.026 |
| HMDB0246022 | 3,4-Diaminopyridine | POS | 1.917 | 0.035 | 1.696 | 0.041 |
| HMDB0247060 | 2-Amino-3-(2-fluoro-3,4-dihydroxyphenyl)propanoic acid | POS | 0.383 | 0.017 | 2.492 | 0.030 |
| HMDB0247315 | (1R,2R)-2-Amino-1-(4-nitrophenyl)propane-1,3-diol | POS | 0.807 | 0.010 | 1.157 | 0.027 |
| HMDB0247746 | Abeado | POS | 1.657 | 0.037 | 1.673 | 0.041 |
| HMDB0248124 | Aldicarb | POS | 1.622 | 0.007 | 2.919 | 0.026 |
| HMDB0248237 | alpha-Muramic acid | POS | 1.346 | 0.018 | 1.748 | 0.030 |
| HMDB0248980 | Cyclohexa-1,2,4,5-tetraene | POS | 0.796 | 0.021 | 1.193 | 0.032 |
| HMDB0249006 | Benzo[d]isoxazol-3-ol | POS | 0.190 | 0.034 | 2.212 | 0.040 |
| HMDB0249237 | Bis-A-tda | POS | 0.805 | 0.049 | 1.008 | 0.049 |
| HMDB0249399 | 2-(2-Cyclohexylethylhydrazono)propionic acid | POS | 0.465 | 0.011 | 1.943 | 0.027 |
| HMDB0249626 | Apricoxib | POS | 0.777 | 0.031 | 1.074 | 0.039 |
| HMDB0250132 | Chlorphentermine | POS | 0.742 | 0.001 | 1.425 | 0.010 |
| HMDB0251354 | Diisopropanolamine | POS | 0.511 | 0.021 | 1.842 | 0.031 |
| HMDB0252802 | 2-Pentenedial | POS | 0.746 | 0.001 | 1.346 | 0.009 |
| HMDB0253648 | Isoindoline | POS | 0.814 | 0.002 | 1.195 | 0.012 |
| HMDB0255409 | 3-(5-Methoxy-4-oxocyclohex-2-en-1-yl)prop-2-enoic acid | POS | 1.268 | 0.036 | 1.539 | 0.041 |
| HMDB0255640 | 1-Nitrobenzantrone | POS | 0.111 | 0.019 | 2.237 | 0.030 |
| HMDB0255800 | Tropolone A | POS | 1.546 | 0.012 | 2.852 | 0.027 |
| HMDB0255909 | Octinoxate | POS | 1.262 | 0.032 | 1.569 | 0.039 |
| HMDB0255957 | Olodaterol | POS | 0.681 | 0.003 | 1.483 | 0.017 |
| HMDB0256111 | Panipenem | POS | 0.594 | 0.033 | 1.695 | 0.040 |
| HMDB0256927 | Puromycin aminonucleoside | POS | 1.255 | 0.037 | 1.354 | 0.041 |
| HMDB0257744 | 9-(1,3-Dioxolan-2-yl)purine-2,6-diamine | POS | 0.258 | 0.011 | 2.065 | 0.027 |
| HMDB0258539 | Succinamide | POS | 0.425 | 0.006 | 2.253 | 0.026 |
| HMDB0258918 | tetramethylmelamine | POS | 0.302 | 0.011 | 1.914 | 0.026 |
| HMDB0259017 | Thiomorpholine | POS | 0.656 | < 0.001 | 1.769 | 0.017 |
| HMDB0259246 | TriMM | POS | 0.411 | 0.007 | 1.772 | 0.025 |
| HMDB0259631 | 7-beta-Hydroxyepiandrosterone | POS | 0.612 | 0.018 | 1.518 | 0.030 |
| LMFA05000709 | 5,6-undecadien-8,10-diyn-1-ol | POS | 1.221 | 0.033 | 1.265 | 0.039 |
| LMFA08020239 | N-(3-oxo-octanoyl)-homoserine thiolactone | POS | 0.814 | 0.007 | 1.066 | 0.025 |
| LMSP01040003 | C17 Sphinganine | POS | 0.071 | 0.022 | 2.403 | 0.033 |
| B100019 | Itaconic acid | NEG | 0.827 | 0.007 | 1.082 | 0.024 |
| B100056 | L-Tryptophan | NEG | 0.769 | < 0.001 | 1.404 | 0.014 |
| B100279 | L-Proline | NEG | 0.740 | 0.017 | 1.125 | 0.030 |
| B100397 | Sebacic acid | NEG | 0.732 | 0.012 | 1.351 | 0.026 |
| B200014 | p-Cresol sulfate | NEG | 0.377 | 0.006 | 2.846 | 0.025 |
| B200042 | Phenyllactic acid | NEG | 0.543 | < 0.001 | 1.961 | 0.011 |
| B200047 | Hydroxyphenyllactic acid | NEG | 0.717 | 0.016 | 1.233 | 0.030 |
| B300211 | 5-Aminovaleric acid | NEG | 0.687 | 0.005 | 1.395 | 0.022 |
| B300283 | 3-[3-(Sulfooxy)phenyl]propanoic acid | NEG | 0.038 | 0.035 | 3.169 | 0.041 |
| B300308 | 1-[(5-Amino-5-carboxypentyl)amino]-1-deoxyfructose | NEG | 1.522 | 0.016 | 2.103 | 0.029 |
| B300536 | 2,3-Dihydroxy-5-methylthio-4-pentenoic acid | NEG | 0.529 | 0.012 | 1.701 | 0.026 |
| 1.1_367.10529 | Cnidioside A | NEG | 1.418 | 0.010 | 1.743 | 0.027 |
| 6.767_407.20774 | NP-020353 | NEG | 0.309 | 0.012 | 1.973 | 0.026 |
| 6.804_395.16334 | benzyl 4-{[(1,3-benzodioxol-5-ylmethyl) amino] carbonyl} tetrahydro-1  (2H)- pyridinecarboxylate | NEG | 0.007 | 0.042 | 2.535 | 0.044 |
| B100328 | trans-Cinnamic acid | NEG | 0.691 | < 0.001 | 1.594 | 0.011 |
| B200013 | Indoleacrylic acid | NEG | 0.575 | 0.037 | 1.675 | 0.041 |
| B200058 | O-methoxycatechol-O-sulphate | NEG | 0.669 | 0.026 | 1.299 | 0.036 |
| B300052 | 3-[4-(sulfooxy)phenyl]propanoic acid | NEG | 0.512 | 0.004 | 1.718 | 0.021 |
| B300778 | Losartan | NEG | 0.708 | 0.007 | 1.317 | 0.024 |
| Reference-408 | DL-Arginine | NEG | 0.778 | 0.017 | 1.072 | 0.029 |
| Reference-550 | 4-Phenolsulfonic acid | NEG | 1.668 | 0.010 | 1.869 | 0.026 |
| 0.729_375.13842 | N1-[4-(aminosulfonyl)phenyl]-2-[4-(tert-butyl)phenoxy]propanamide | NEG | 1.531 | 0.008 | 1.983 | 0.025 |
| 0.984_328.10706 | NP-022371 | NEG | 0.390 | 0.001 | 2.463 | 0.009 |
| 2.555_218.09332 | Methyl 2,5-dimethyl-4-(1H-pyrazol-3-yl)-1H-pyrrole-3-carboxylate | NEG | 0.293 | 0.004 | 2.192 | 0.020 |
| 4.608_194.08222 | L-Tyrosine methyl ester | NEG | 0.471 | 0.018 | 2.153 | 0.030 |
| 4.884_210.07712 | 2-Amino-3-(4-hydroxy-3-methoxyphenyl)propanoic acid | NEG | 0.065 | 0.001 | 3.353 | 0.009 |
| 6.804_449.21805 | Nandrolone glucuronide | NEG | 0.026 | 0.047 | 2.046 | 0.048 |
| B200059 | 4-Methylcatechol 2-sulfate | NEG | 0.657 | 0.003 | 1.456 | 0.016 |
| Reference-1498 | D-Saccharic acid | NEG | 0.809 | 0.015 | 1.078 | 0.028 |
| Reference-2560 | N-Acetyl-D-galactosamine | NEG | 1.569 | 0.007 | 2.535 | 0.023 |
| Reference-7172 | Prednisolone 21-hemisuccinate | NEG | 1.725 | 0.012 | 2.947 | 0.025 |
| Reference-7795 | 3,3'-Diisopropyl-6,6'-dimethyl-2,2',5,5'-biphenyltetrol | NEG | 0.020 | 0.046 | 2.171 | 0.048 |
| Reference-8706 | 5-Amino-3-(4-methoxyphenyl)-5-oxopentanoic acid | NEG | 0.761 | 0.006 | 1.236 | 0.025 |
| Reference-8914 | 1. (1-Amino-1-oxo-2-propanyl)-1,3,4-trihydroxy-5-{[(4-methylphenyl) 2. carbamoyl]amino}cyclohexanecarboxamide | NEG | 0.706 | 0.008 | 1.408 | 0.024 |
| C00916 | Cephalosporin C | NEG | 1.539 | 0.012 | 2.117 | 0.025 |
| C02928 | 2-Dehydro-D-xylonate | NEG | 0.825 | 0.016 | 1.012 | 0.029 |
| C18584 | Quinoclamin | NEG | 0.323 | < 0.001 | 2.849 | 0.004 |
| HMDB0000167 | L-Threonine | NEG | 0.777 | 0.002 | 1.241 | 0.012 |
| HMDB0000491 | 3-Methyl-2-oxovaleric acid | NEG | 0.724 | 0.040 | 1.045 | 0.043 |
| HMDB0013974 | 4'-Hydroxydiclofenac | NEG | 0.685 | 0.016 | 1.374 | 0.029 |
| HMDB0242256 | (+)-Lysergic acid | NEG | 0.160 | 0.001 | 3.099 | 0.008 |
| HMDB0243964 | 1-Naphthylamine | NEG | 0.572 | 0.038 | 1.687 | 0.042 |
| HMDB0244558 | N-(p-Toluenesulfonyl)-L-phenylalanine | NEG | 1.333 | 0.015 | 1.599 | 0.028 |
| HMDB0244969 | 1-(10H-Phenothiazin-2-yl)ethanone | NEG | 0.576 | 0.008 | 1.604 | 0.024 |
| HMDB0246568 | 4-Phenyl-1,2,4-triazoline-3,5-dione | NEG | 0.717 | 0.020 | 1.195 | 0.031 |
| HMDB0252496 | Fructosyl valine | NEG | 1.439 | 0.023 | 2.210 | 0.034 |
| HMDB0254559 | methyl (Z)-6-(3,7-dimethyl-2-oxo-1,3-benzoxazol-5-yl)-6-(3-methoxy-7-  methyl-1,2-benzoxazol-5-yl)hex-5-enoate | NEG | 1.673 | 0.013 | 2.938 | 0.026 |
| HMDB0255498 | 5'-N-Ethylcarboxamidoadenosine | NEG | 1.709 | 0.004 | 2.707 | 0.020 |
| HMDB0257777 | [(2R,3S,4R,5R)-3,4,5,6-Tetrahydroxy-1-oxohexan-2-yl] (2S)-2-aminopropanoate | NEG | 1.434 | 0.011 | 2.289 | 0.026 |
| HMDB0258093 | Dicarbamoyl (2S)-2-aminopentanedioate | NEG | 0.607 | 0.004 | 1.551 | 0.019 |
| HMDB0258590 | Sulfoacetic acid | NEG | 0.784 | 0.022 | 1.137 | 0.032 |
| HMDB0258964 | Theodrenaline | NEG | 2.187 | 0.049 | 2.416 | 0.049 |
| HMDB0259526 | 2-Propanamine, N,N,2-trimethyl-1-((3-phenyl-2-quinolinyl)thio) | NEG | 1.576 | 0.007 | 2.423 | 0.023 |
| LMFA01020407 | 3-Hydroxy-11-methyl-stearic acid | NEG | 0.410 | 0.019 | 1.908 | 0.030 |

HMC= high myopic cataract; ARC= age-related cataract; VIP= Variable importance in the projection.

**Table S5. Pathways that were identified by KEGG analysis based on aqueous humor metabolites of high myopic cataract patients.**

| **Pathway** | **Count** | **P value** | **FDR** | **KEGG Names** |
| --- | --- | --- | --- | --- |
| Protein digestion and absorption | 6 | < 0.001 | < 0.001 | L-Tryptophan;L-Phenylalanine;L-Proline;4-Ethoxybenzaldehyde;Indole;L-Aspartic acid |
| Aminoacyl-tRNA biosynthesis | 5 | < 0.001 | < 0.001 | L-Tryptophan;L-Phenylalanine;L-Proline;4-Ethoxybenzaldehyde;L-Aspartic acid |
| D-Amino acid metabolism | 6 | < 0.001 | < 0.001 | L-Phenylalanine;L-Proline;4-Ethoxybenzaldehyde;5-Aminovaleric acid;D-(+)-Proline;L-Aspartic acid |
| Central carbon metabolism in cancer | 5 | < 0.001 | < 0.001 | L-Tryptophan;L-Phenylalanine;L-Proline;4-Ethoxybenzaldehyde;L-Aspartic acid |
| Biosynthesis of amino acids | 5 | < 0.001 | 0.001 | L-Tryptophan;L-Phenylalanine;L-Proline;4-Ethoxybenzaldehyde;L-Aspartic acid |
| Arginine and proline metabolism | 4 | < 0.001 | 0.001 | L-Proline;4-Ethoxybenzaldehyde;5-Aminovaleric acid;D-(+)-Proline |
| Mineral absorption | 3 | < 0.001 | 0.001 | L-Tryptophan;L-Phenylalanine;L-Proline |
| Tryptophan metabolism | 4 | < 0.001 | 0.001 | L-Tryptophan;Indole;L-5-Hydroxytryptophan;Skatole |
| Phenylalanine, tyrosine and tryptophan biosynthesis | 3 | < 0.001 | 0.002 | L-Tryptophan;L-Phenylalanine;Indole |
| Neuroactive ligand-receptor interaction | 3 | < 0.001 | 0.004 | 4-Ethoxybenzaldehyde;L-Aspartic acid;Cortisol |
| ABC transporters | 4 | < 0.001 | 0.004 | L-Phenylalanine;L-Proline;4-Ethoxybenzaldehyde;L-Aspartic acid |
| 2-Oxocarboxylic acid metabolism | 4 | < 0.001 | 0.005 | L-Tryptophan;L-Phenylalanine;4-Ethoxybenzaldehyde;L-Aspartic acid |
| Arginine biosynthesis | 2 | 0.002 | 0.010 | 4-Ethoxybenzaldehyde;L-Aspartic acid |
| Alanine, aspartate and glutamate metabolism | 2 | 0.004 | 0.020 | 4-Ethoxybenzaldehyde;L-Aspartic acid |
| Serotonergic synapse | 2 | 0.008 | 0.039 | L-Tryptophan;L-5-Hydroxytryptophan |
| Glycine, serine and threonine metabolism | 2 | 0.009 | 0.041 | L-Tryptophan;L-Aspartic acid |
| Histidine metabolism | 2 | 0.010 | 0.044 | 4-Ethoxybenzaldehyde;L-Aspartic acid |
| FoxO signaling pathway | 1 | 0.013 | 0.052 | 4-Ethoxybenzaldehyde |
| Nicotinate and nicotinamide metabolism | 2 | 0.014 | 0.053 | 3-Succinoylpyridine;L-Aspartic acid |
| Biosynthesis of cofactors | 4 | 0.014 | 0.052 | L-Tryptophan;Caprylic acid;4-Ethoxybenzaldehyde;L-Aspartic acid |
| Huntington disease | 1 | 0.016 | 0.056 | 4-Ethoxybenzaldehyde |
| Spinocerebellar ataxia | 1 | 0.016 | 0.053 | 4-Ethoxybenzaldehyde |
| Long-term potentiation | 1 | 0.020 | 0.061 | 4-Ethoxybenzaldehyde |
| Lipoic acid metabolism | 1 | 0.020 | 0.059 | Caprylic acid |
| Phospholipase D signaling pathway | 1 | 0.020 | 0.056 | 4-Ethoxybenzaldehyde |
| African trypanosomiasis | 1 | 0.023 | 0.063 | L-Tryptophan |
| Cocaine addiction | 1 | 0.023 | 0.061 | 4-Ethoxybenzaldehyde |
| Nicotine addiction | 1 | 0.023 | 0.059 | 4-Ethoxybenzaldehyde |
| Aldosterone-regulated sodium reabsorption | 1 | 0.023 | 0.057 | Cortisol |
| Glutamatergic synapse | 1 | 0.023 | 0.055 | 4-Ethoxybenzaldehyde |
| Long-term depression | 1 | 0.023 | 0.053 | 4-Ethoxybenzaldehyde |
| Circadian entrainment | 1 | 0.026 | 0.059 | 4-Ethoxybenzaldehyde |
| GABAergic synapse | 1 | 0.029 | 0.064 | 4-Ethoxybenzaldehyde |
| Gap junction | 1 | 0.029 | 0.062 | 4-Ethoxybenzaldehyde |
| Amphetamine addiction | 1 | 0.029 | 0.060 | 4-Ethoxybenzaldehyde |
| Prostate cancer | 1 | 0.032 | 0.065 | Cortisol |
| Alcoholism | 1 | 0.032 | 0.063 | 4-Ethoxybenzaldehyde |
| Amyotrophic lateral sclerosis | 1 | 0.036 | 0.067 | 4-Ethoxybenzaldehyde |
| Synaptic vesicle cycle | 1 | 0.036 | 0.066 | 4-Ethoxybenzaldehyde |
| Sphingolipid metabolism | 1 | 0.036 | 0.064 | Phytosphingosine |
| Cortisol synthesis and secretion | 1 | 0.039 | 0.068 | Cortisol |
| Fatty acid biosynthesis | 1 | 0.038 | 0.066 | Caprylic acid |
| Cushing syndrome | 1 | 0.042 | 0.070 | Cortisol |
| Retrograde endocannabinoid signaling | 1 | 0.049 | 0.079 | 4-Ethoxybenzaldehyde |
| Carbon metabolism | 2 | 0.054 | 0.087 | 4-Ethoxybenzaldehyde;L-Aspartic acid |
| Nitrogen metabolism | 1 | 0.055 | 0.085 | 4-Ethoxybenzaldehyde |
| Proximal tubule bicarbonate reclamation | 1 | 0.055 | 0.083 | 4-Ethoxybenzaldehyde |
| Pathways of neurodegeneration - multiple diseases | 1 | 0.066 | 0.100 | 4-Ethoxybenzaldehyde |
| Taurine and hypotaurine metabolism | 1 | 0.076 | 0.112 | 4-Ethoxybenzaldehyde |
| Pathways in cancer | 1 | 0.082 | 0.118 | Cortisol |
| Pantothenate and CoA biosynthesis | 1 | 0.088 | 0.125 | L-Aspartic acid |
| Glutathione metabolism | 1 | 0.091 | 0.126 | 4-Ethoxybenzaldehyde |
| Ferroptosis | 1 | 0.091 | 0.124 | 4-Ethoxybenzaldehyde |
| beta-Alanine metabolism | 1 | 0.10 | 0.134 | L-Aspartic acid |
| Taste transduction | 1 | 0.10 | 0.131 | 4-Ethoxybenzaldehyde |
| Galactose metabolism | 1 | 0.138 | 0.177 | N-Acetyl-D-galactosamine |
| Lysine degradation | 1 | 0.138 | 0.174 | 5-Aminovaleric acid |
| Butanoate metabolism | 1 | 0.141 | 0.175 | 4-Ethoxybenzaldehyde |
| Phenylalanine metabolism | 1 | 0.149 | 0.182 | L-Phenylalanine |
| Ascorbate and aldarate metabolism | 1 | 0.168 | 0.203 | D-Saccharic acid |
| Glyoxylate and dicarboxylate metabolism | 1 | 0.187 | 0.222 | 4-Ethoxybenzaldehyde |
| Cysteine and methionine metabolism | 1 | 0.193 | 0.224 | L-Aspartic acid |
| Tyrosine metabolism | 1 | 0.222 | 0.254 | 3,4-Dihydroxyphenylpropionic acid |
| Neomycin, kanamycin and gentamicin biosynthesis | 1 | 0.222 | 0.250 | 4-Ethoxybenzaldehyde |
| Chemical carcinogenesis - DNA adducts | 1 | 0.227 | 0.252 | 3-Succinoylpyridine |
| Drug metabolism - cytochrome P450 | 1 | 0.249 | 0.273 | Indoleacrylic acid |
| Bile secretion | 1 | 0.264 | 0.284 | Cortisol |
| Steroid hormone biosynthesis | 1 | 0.279 | 0.295 | Cortisol |
| Amino sugar and nucleotide sugar metabolism | 1 | 0.304 | 0.318 | N-Acetyl-D-galactosamine |
| Metabolism of xenobiotics by cytochrome P450 | 1 | 0.329 | 0.339 | 3-Succinoylpyridine |
| Porphyrin metabolism | 1 | 0.349 | 0.354 | 4-Ethoxybenzaldehyde |
| Biosynthesis of nucleotide sugars | 1 | 0.484 | 0.485 | N-Acetyl-D-galactosamine |
