# Supplementary material for: Integrative multi-omics identifies coordinated alterations in the gut microbiome and in plasma and aqueous humor metabolomes in high myopic cataract
Source: Sci Rep. 2025 Nov 12;15:39636. doi: 10.1038/s41598-025-23265-8 (PMC12612057; doi:10.1038/s41598-025-23265-8)
Supplement: Supplementary file 2 — Supplementary Material 2 [file 41598_2025_23265_MOESM2_ESM.docx]

**Supplementary Methods**

**Feces specimens, plasma specimens, and** **aqueous humor specimens collection**

All the eligible patients collected their feces specimens into sterile stool samplers according to concomitant instructions. Most of the specimens were stored at 4 °C for less than 6 h and then quickly transferred to -80 °C until use.

Plasma specimens were obtained from fasting blood samples that were anticoagulated with EDTA-K2. All the samples were centrifuged 1500 rpm, for 10 min. About 1.5 mL plasma specimens were collected into freezing tubes and stored at -80 °C until use.

After receiving local anesthesia, all patients underwent cataract phacoemulsification with IOL implantation surgery. Self-sealing 2.4 mm superior corneal incisions were conducted for all patients. The aqueous humor (AH) specimens (100-150ul) were immediately collected with a 1ml injector with a 30-gauge needle from each patient. The AH specimens were stored at -80 °C with 0.5 mL freezing tubes.

**Analysis of fecal gut microbiome**

The DNA was extracted using the magnetic bead-based DNA extraction method. DNA was transferred to 1.5 mL centrifuge tube. Then the V3-V4 region of 16S rDNA was detected. After quality inspection, 30 ng DNA of qualified specimens was taken to be involved in Polymerase chain reaction (PCR) amplification. The barcoded primers were 338F 5’-ACTCCTACGGGAGGCAGCAG-3’ and 806R 5’-GGACTACHVGGGTWTCTAAT. Agencourt AMPure XP magnetic beads were used to purify PCR amplicons. Selected amplicons were dissolved in the elution buffer. The DNA library was then detected using Agilent 2100 Bioanalyzer.

The Raw data were filtered to generate high-quality clean reads as follows using iTools Fqtools fqcheck (v.0.25), cutadapt (v.2.6) and readfq (v1.0): (1) Truncate reads whose average phred quality values are lower than 20 over a 30 bp sliding window will be truncated. Remove reads whose lengths are 75% of their original lengths after truncation. (2) Remove reads that are contaminated by adapter sequences. (3) Remove reads with ambiguous base (N base). (4) Remove low complexity reads.

Tags similarity over 97% were assigned to the same operational taxonomic units (OTUs) via a clustering process conducted in USEARCH (usearch11.0.667_i86). OTU representative sequences are aligned against the database for taxonomic annotation by RDP classifer (version 2.2) software (sequence identity is set to be 0.6). Mothur(version.1.31.2) and QIIME(version 1.80) were performed to obtain the results of alpha diversity analysis and beta diversity analysis respectively. Linear discriminant analysis effect size (LEfSe) analysis was used to identify distinct taxa at the class, order, family, and genus level.

**Meta****bolomic analysis of** **plasma and** **aqueous humor**

Each 100 µL Plasma specimen and aqueous humor specimen was added 700 µL extract solution (methanol: acetonitrile: water= 4: 2: 1, the internal standard mixed as well), and the sample was oscillated for 1 minute and placed in the refrigerator at -20 °C for 2 h, then centrifuged at 4 °C, 25000 rpm, for 15 min. About 600 µL supernatant was transferred into a new Eppendorf tube for extractor drying. After that 180 µL methanol: purified water (1:1) was added to the sample and vortex-oscillated for 10 min. The supernatant of each sample was transferred into a new Eppendorf tube after centrifuging at 4 °C, 25000 rpm, for 15 min. Mixing 20 µL supernatant from each sample to complete the preparation of the final quality-controlled (QC) samples. Metabolites of plasma and aqueous humor QC samples were measured using LC-MS/MS analysis in Waters 2777C UPLC（Waters, USA）and Q-Exactive HF high-resolution mass spectrometer (Thermo Fisher Scientific, USA) platforms. The BEH C18 column (1.7 μm 2.1x100 mm, Waters, USA) was used in the chromatographic process at a flow rate of 0.35 mL/min. The positive eluents were 0.1% formic acid in water (eluent A) and 0.1% formic acid in methanol (eluent B). The negative eluents were 10mM ammonium formate in water (eluent A) and 10mM ammonium formate in 95% methanol (eluent B). First and second-level mass spectrometry data collection was conducted. The condition of the mass spectrometer was set as follows：

spray voltage was 3.8 kV (positive polarity mode) and 3.2 kV (negative polarity mode), the aux gas flow rate was 10 arb, the sheath gas flow rate was 40 arb, the capillary temperature was 320 °C, the aux gas heater temperature was 350 °C. The data were then imported into Compound Discoverer 3.3 software (Thermo Fisher Scientific, USA). By pairing with the BGI Metabolome Database (BMDB), mzCloud database, and ChemSpider database, metabolites were identified finally.

Metabolomic analysis was performed on the “Dr. Tom” platform which was developed by BGI Genomics Co.,Ltd.(Shen Zhen, China). Fold change was obtained by the mean and standard deviation (SD) of each metabolite. When detecting the distribution difference in metabolites of the two groups, k-fold cross-validation (k=7, stratified) was used to obtain the partial least squares-discriminant analysis, to verify model robustness and exclude overfitting, we carried out response permutation testing (n = 200). Variable Important for the Projection (VIP) was used to assist in the identification of distinct metabolites (VIP>1 means the metabolite was significant). Metabolites with VIP>1, Fold Change (FC) ≥1.2, and P value <0.05 were identified as significant. In volcano plot, the data of the x-axis equaled log2 (FC), and the y-axis equaled -log10 (P value).

We further explored metabolic pathways using the Kyoto Encyclopedia of Genes and Genomes (KEGG) database. The metabolic pathways with a P value less than 0.05 were statistically significant. The 10 metabolic pathways with the smallest P value were shown in the bubble chart. The diﬀerential abundance scores of these 10 metabolic pathways were then calculated to clarify their influence.

**Statistical analysis**

Statistical analysis was performed using R studio (version 4.2.2) and StataSE15 (version 15.0, Stata Corp LP, TX, USA). All continuous variables were expressed as means ± standard deviations (SD). Categorical variables were counted as values and percentages. The Shapiro-Francia W’ test was used to confirm the normal distribution. Student's t-tests were used to assess the difference in ocular parameters between age-related cataract patients (ARC) and high myopic cataract patients (HMC). A two-sided P-value <0.05 was considered statistically significant. We also applied the Benjamini–Hochberg false discovery rate (FDR) procedure. Both raw p-values and FDR are reported. Associations with FDR < 0.05 were considered still statistically significant, while associations with 0.05 ≤ FDR < 0.10 were considered suggestive.

The relationship between the distinct gut taxa and distinct metabolites in plasma and aqueous humor was explored via Spearman correlation analysis using the “corrplot” package. We extracted the 10 metabolites with the largest FC and P values less than 0.05. Then paired with distinct gut taxa, using the “corrplot” package obtain the correlation coefficient and p value.

Correlation between clinical parameters and distinct gut microbiome, as well as the top 10 metabolites, were evaluated by Spearman correlation analysis using the “corrplot” package. The “pROC” package was used to analyze the receiver-operating characteristic (ROC) curve and the area under the ROC curves (AUC) between two groups to figure out distinct metabolomic biomarkers.
