# Supplementary material for: Integrative multi-omics identifies coordinated alterations in the gut microbiome and in plasma and aqueous humor metabolomes in high myopic cataract
Source: Sci Rep. 2025 Nov 12;15:39636. doi: 10.1038/s41598-025-23265-8 (PMC12612057; doi:10.1038/s41598-025-23265-8)
Supplement: Supplementary file 3 — Supplementary Material 3 [file 41598_2025_23265_MOESM3_ESM.pdf]

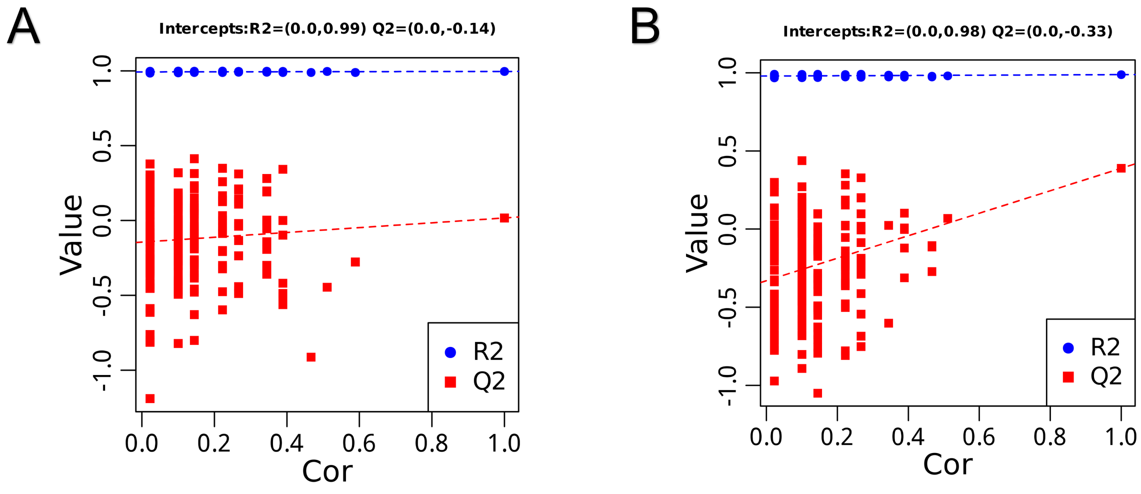

Fig. S1 (A) Response permutation testing of the PLS-DA model of plasma specimens. (B) Response permutation testing of the PLS-DA model of aqueous humor specimens.
